# Antimicrobial resistance in Africa: a systematic review

**DOI:** 10.1186/s12879-017-2713-1

**Published:** 2017-09-11

**Authors:** Birkneh Tilahun Tadesse, Elizabeth A. Ashley, Stefano Ongarello, Joshua Havumaki, Miranga Wijegoonewardena, Iveth J. González, Sabine Dittrich

**Affiliations:** 1 Foundation for Innovative New Diagnostics (FIND), Campus Biotech Building B2 Level 0, 9 Chemin des Mines, 1202 Geneva, Switzerland; 2 College of Medicine and Health Sciences, Department of Pediatrics, Hawassa University, Hawassa, Ethiopia; 3 Special Programme for Research & Training in Tropical Diseases (TDR), World Health Organization, Avenue Appia 20, 1211, 27 Geneva, Switzerland; 4 Myanmar-Oxford Clinical Research Unit (MOCRU), Yangon, Myanmar

**Keywords:** Antimicrobial resistance, Africa, Bacteria, Systematic review

## Abstract

**Background:**

Antimicrobial resistance (AMR) is widely acknowledged as a global problem, yet in many parts of the world its magnitude is still not well understood. This review, using a public health focused approach, aimed to understand and describe the current status of AMR in Africa in relation to common causes of infections and drugs recommended in WHO treatment guidelines.

**Methods:**

PubMed, EMBASE and other relevant databases were searched for recent articles (2013–2016) in accordance with the PRISMA guidelines. Article retrieval and screening were done using a structured search string and strict inclusion/exclusion criteria. Median and interquartile ranges of percent resistance were calculated for each antibiotic-bacterium combination.

**Results:**

AMR data was not available for 42.6% of the countries in the African continent. A total of 144 articles were included in the final analysis. 13 Gram negative and 5 Gram positive bacteria were tested against 37 different antibiotics. Penicillin resistance in *Streptococcus pneumoniae* was reported in 14/144studies (median resistance (MR): 26.7%). Further 18/53 (34.0%) of *Haemophilus influenza* isolates were resistant to amoxicillin. MR of *Escherichia coli* to amoxicillin, trimethoprim and gentamicin was 88.1%, 80.7% and 29.8% respectively. Ciprofloxacin resistance in Salmonella Typhi was rare. No documented ceftriaxone resistance in *Neisseria gonorrhoeae* was reported, while the MR for quinolone was 37.5%. Carbapenem resistance was common in *Acinetobacter* spp. and *Pseudomonas aeruginosa* but uncommon in *Enterobacteriaceae*.

**Conclusion:**

Our review highlights three important findings. First, recent AMR data is not available for more than 40% of the countries. Second, the level of resistance to commonly prescribed antibiotics was significant. Third, the quality of microbiological data is of serious concern. Our findings underline that to conserve our current arsenal of antibiotics it is imperative to address the gaps in AMR diagnostic standardization and reporting and use available information to optimize treatment guidelines.

**Electronic supplementary material:**

The online version of this article (10.1186/s12879-017-2713-1) contains supplementary material, which is available to authorized users.

## Background

Internationally, there is a growing concern over antimicrobial resistance (AMR) which is currently estimated to account for more than 700,000 deaths per year worldwide [1]. If no appropriate measures are taken to halt its progress, AMR will cost approximately 10 million lives and about US$100 trillion per year by 2050 [1]. In contrast to some other health issues, AMR is a problem that concerns every country irrespective of its level of income and development as resistant pathogens do not respect borders [1, 2].

Despite the threat presented by AMR, the 2014 World Health Organization (WHO) and the recent O’Neill report describe significant gaps in surveillance, standard methodologies and data sharing [1, 2]. The 2014 WHO report identified Africa and South East Asia as the regions without established AMR surveillance systems [2]. This lack of quality data is problematic often leading to treatment guidelines that are not adequate for the local situation. The gap in public health capacity is also an issue given the changing resistance mechanisms and the emergence of multidrug-resistant bacteria that can only be detected through systematic screening in quality assured microbiology laboratories [3, 4].

One factor contributing to AMR is misuse of antibiotics. Improved malaria diagnostics and the recognition that malaria transmission is decreasing globally has highlighted the lack of tests for other infections and many patients who test negative for malaria are treated with antibiotics indiscriminately [5–7]. Clinical treatment algorithms like the Integrated Management of Neonatal and Childhood Illnesses (IMNCI) and Integrated Management of Adolescent and Adulthood Illnesses (IMAI) guidelines implemented by the WHO have tried to optimize antibiotic prescription in resource-limited settings, however overuse of antibiotics is still happening [8–11]. Following these guidelines amoxicillin or sulfamethoxazole/trimethoprim are the first line drugs for urinary tract infections (UTI) or acute respiratory tract infections. A combination of ampicillin and gentamicin or ceftriaxone are the drugs of choice for treating blood stream infections (BSI) and sulfamethoxazole/trimethoprim or ciprofloxacin are recommended for the treatment of dysentery [8, 9, 12].

A number of recent reviews summarized AMR data in Africa, most recently Leopold et al. (2014) which focused on Sub-Saharan Africa. The authors found a high level of resistance to the commonly used antibiotics in the sub-Saharan African region. For example, 90% of Gram negatives were resistant to chloramphenicol, a commonly used antibiotic. In contrast, resistance to third-generation cephalosporins (like ceftriaxone) was less common, recommending this group for use [13].

To design suitable local and global interventions, it is important to understand the current status of AMR and identify knowledge gaps. The purpose of this review is to summarize the available information about the occurrence of AMR on the entire African continent and describe laboratory methods currently in use, to identify knowledge gaps and highlight diagnostic needs.

## Methods

### Search strategy

PubMed, EMBASE, Science Daily, the Cochrane Database for Systematic Reviews, African Journals Online Library and Free-text Web Searches using Google Scholar were searched for articles published in English from January 1, 2013 through January 31, 2016. Literature before January 2013 was covered in previous reviews [13, 14]. Reference lists of relevant articles were checked for additional titles for inclusion in the review. Key words used for the search were “*Antimicrobial Resistance*”, “*Antimicrobial Susceptibility*”, “*Surveillance*”, “*Diagnostic*”, “*Africa*” and specific names of all African countries. The detailed search strategy, as well as details of the article quality assessment can be found in Additional file 1.

### Selection criteria

Articles reporting AMR prevalence, availability of AMR surveillance systems or diagnostic needs of antibiotic resistance in the whole African region were included. Based on the abstract, articles of all types with any data on etiology and antibiotic susceptibility pattern were included for further screening. Studies were included or excluded following predefined criteria.

Inclusion:

- Reports on AMR in humans from the African region
- Abstracts and full text available in English
- Drug sensitivity testing done in a laboratory setting with defined cutoffs for drug susceptibility testing
- The denominator as total isolates clearly described for population based studies
- Case reports and case series

Exclusion:

- Reports published before 2013
- Studies only focused on malaria, HIV or tuberculosis without AMR information
- Studies without information on total studied isolates

### Selection procedure

Titles and abstracts of all the articles retrieved through the search were screened. In the event of uncertainty as to whether articles met the criteria for study inclusion they were discussed with two co- authors. Articles selected for full text review were obtained using PubMed, WHO GIFT access, HINARI, institutional websites or by contacting the authors directly. Names of authors from articles in the search results were not blinded for abstract or full-text review.

### Data extraction

Data extraction was done using a predesigned and pretested database, developed for the purposes of this review using Microsoft Excel 2013. Information extracted included article information (PMID, first author, year of publication, duration of data collection and country), study design (sample size, age group, hospital acquired or community acquired, number of specimens collected, and clinical syndrome), pathogen identification and antimicrobial susceptibility testing methodology, laboratory accreditation information and antibacterial resistance data.

### Article quality assessment

The quality of each article was assessed using a tool modified for the purposes of this study from criteria published by Omulo et al. and the Cochrane guidelines for assessing bias in observational studies. Since a limited number of articles was available, results of the quality assessment were not used for inclusion/exclusion. The quality criteria included 26 items to assess the design, details of sample collection, processing and storage, reporting on AMR methodologies and quality assurance strategies.

### Data analysis

We calculated prevalence, median resistance (MR) and inter quartile range (IQR) of resistance for each bacterium-antibiotic combination to calculate a standardized measure from the collected data. Pediatric age was considered less than 18 years and neonatal age less than 28 days. Meta-analysis was not conducted because of the large variability in AMR methodology, geography and the small number of articles available per country. Since the number of studies from hospital/in-patient settings was small, they were combined and median percentages with interquartile ranges were generated. Statistical analyses and visualization were performed using Microsoft Excel 2013, STATA v14 (STATA, College Station, TX, USA) and R-software 3.3.1.

## Results

### Data and study characteristics

**Fig. 1:**
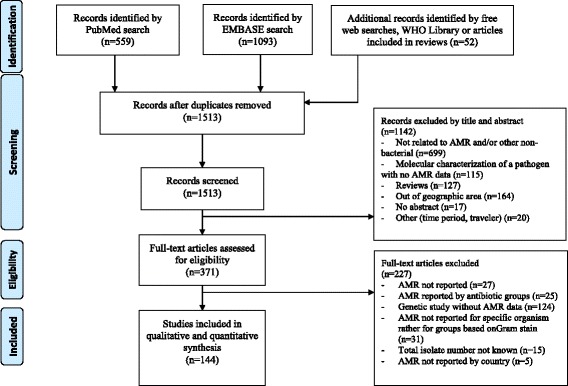
PRISMA Diagram of the article selection procedure for articles published between January 2013 and January 2016.The review has been registered to the PROSPERO database of systematic reviews on March 1, 2016 (http://www.crd.york.ac.uk/PROSPERO/myprospero.php) with ID CRD42016035923

**Fig. 2:**
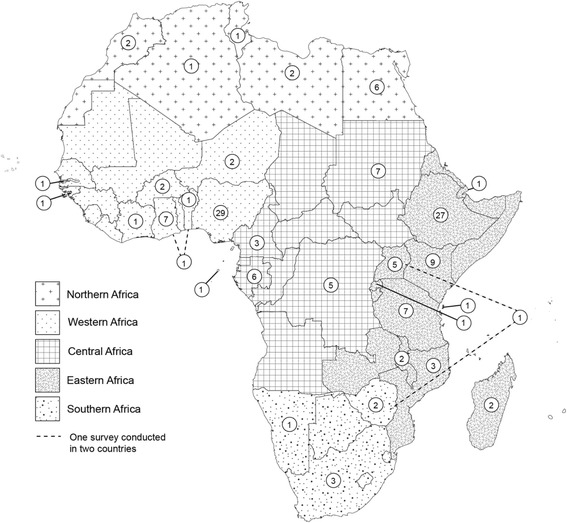
Geographical distribution and number of selected studies between January 2013 and January 2016 in the different African countries. Countries were grouped based on the United Nations Statistics Division classification into Eastern Africa, Southern Africa, Central Africa, Northern Africa and Western Africa

In total, 1704 articles were identified. Of those, 144 studies met the inclusion criteria and were included in the final analysis (Fig. 1). Samples from a total of 149,733 patients were analyzed in the selected studies. The majority of the studies were from East Africa (59/144, 40.9%) while the smallest number of studies were from the South African region (6/144, 4.2%) (Fig. 2). No suitable report was identified from 23/54, 42.6% countries. While the articles were published between 2013 and 2016, the reported data were collected from 1995 to 2015 with the majority from before 2013 (98/144, 68.1%). Most of the studies (92/144, 63.9%) were cross sectional studies or case series (Table 1). Similar numbers of studies were published with susceptibility data for isolates from blood culture (25/144, 17.4%), urine culture (25/144, 17.4%), wound discharge/pus isolates (22/144, 15.3%); and multiple sample types (21/144, 14.6%). More than 80% of the studies fulfilled more than half of the quality parameters (121/144, 84.0%) used to score the articles (Additional file 1). Among the different studies, four different methods for susceptibility testing and five different interpretation guidelines were used (Table 1).

**Table 1:** Characteristics of the articles included in the systematic review

Characteristic	Frequency (%)	References
Publication Year		
2013	67 (46.5)	[17, 19, 26, 43–99] [13, 16, 18, 22, 24, 27, 45, 100–138] [20, 21, 23, 25, 139–172] [173–175]
2014	39 (27.1)	
2015	35 (24.3)	
2016	3 (2.1%)	
End of data collection		NA
Before 2010	17 (11.8)	
Between 2010 and 2013	81 (56.3)	
After 2013	41 (28.5)	
Not mentioned	5 (3.5)	
Study Design		
Cross sectional/Case Series	92 (63.9)	[16–23, 25, 26, 28, 30–32, 36, 38–40, 42, 43, 45, 46, 48–50, 53, 55–58, 60, 63, 67–71, 73–75, 77, 79, 82–85, 87, 89, 90, 92, 93, 95–100, 103–105, 108, 112–116, 119, 123–133, 137, 139–142, 144, 146, 151, 153, 154, 156, 157, 160, 176] [59, 106, 150] [44, 52, 56, 74, 76, 79, 80, 116, 126, 135, 153, 164] [24, 27, 49, 50, 62, 91, 95, 103, 124, 125, 136, 149, 160, 163, 165, 167, 173] [67, 69, 77, 81, 121, 132, 151, 158, 162] [48, 66, 87, 93, 96, 101, 109, 117, 133, 170, 174]
Case control	3 (2.1)	
Prospective Cohort/Clinical Trial	11 (7.6)	
Retrospective	18 (12.5)	
Surveillance	9 (6.3)	
Not Mentioned	11 (7.6)	
Source of Data		
In patient	59 (41.0)	[16, 17, 20, 22, 23, 26, 44–46, 53, 54, 56, 60–62, 64, 66, 68, 71, 72, 77, 80, 84, 88, 90–92, 95, 97, 99, 101, 104, 108, 112, 117–120, 123, 126–129, 132, 135, 136, 138, 147, 148, 153, 155, 158, 162, 164, 166, 167, 173] [18–20, 24, 25, 43, 51, 52, 58, 59, 63, 70, 73–75, 78, 82, 85, 87, 94, 96, 100, 105–107, 111, 113, 116, 117, 131, 133, 134, 139, 140, 142, 150–152, 156, 157, 159, 170–172, 175, 176] [50, 79, 83, 86, 130, 143, 149, 161, 163, 169, 177, 178] [27, 38, 49, 57, 65, 67, 76, 79, 81, 93, 103, 109, 110, 115, 121, 124, 125, 145, 146, 154, 165, 174]
Outpatient	49 (30.0)	
Both	12 (8.3)	
Unknown	24 (17.4)	
Type of Infection		
Hospital Acquired	22 (15.3)	[16, 17, 23, 54, 60–62, 72, 80, 88, 90, 91, 97, 104, 119, 123, 127–129, 135, 147] [18–20, 24, 25, 28, 36, 37, 43, 44, 48, 55, 58–60, 63, 67, 70, 72, 79, 81, 85, 90–92, 96, 98, 101, 102, 116, 118, 119, 124, 125, 127, 135–137, 141, 142, 144, 155–157, 160, 161] [50, 55, 77, 79, 86, 95, 145, 162, 163, 166, 167]
Community acquired	58 (40.3)	
Both	11 (7.6)	
Unable to say	53 (37.5)	
AMR/Drug susceptibility methodology^a^		
Disk Diffusion	118 (81.9)	[38, 56, 63, 74, 81, 84, 86, 96, 104, 115, 140, 146, 148, 150] [65, 77, 116, 121, 132, 155]
VITEK	11 (7.6)	
E-test	2 (1.4)	
MIC	7 (4.9)	
Not mentioned	6 (4.2)	
Laboratory Standard		NA
BSAC	6 4.1)	
CA-SFM/EUCAST	15 (10.4)	
NCCLS/CLSI	105 (72,9)	
Not Mentioned	18 (12.5)	
Organism identification Method^a^		
Morphology	95 (66.0)	[16, 17, 19, 22, 24, 43–45, 50, 51, 53, 54, 56, 58, 62–64, 67, 70, 71, 73, 75, 78, 81–83, 85–87, 89, 90, 92, 94, 95, 97, 98, 101, 103–106, 108, 110–113, 119, 120, 125–129, 131, 134, 138, 139, 142, 151, 152, 155, 157, 162, 164, 165, 169, 172, 174–176] [55, 68, 114, 116, 117, 123, 135, 145, 147–149]
API®	17 (11.8)	
VITEK	6 (4.2)	
NAAT	3 (2.1)	
MALDI-ToF	2 (1.4)	
Not mentioned	21 (14.6)	
Lab Accreditation/Quality assurance activities		
ISO	5 (3.5)	[38, 74, 88, 169, 179] [74, 92, 116, 136, 145, 166]
EQA	6 (4.2)	
Not mentioned	133 (92.3)	
Age Group		NA
Adults	42 (29.2)	
Pediatrics and neonates^b^	34 (23.6)	
Both adults and pediatrics	42 (29.2)	
Neonates only	4 (2.8)	
Unknown	22 (15.3)	

*NA* not applicable, *BSAC* British Society for Antimicrobial Chemotherapy, *CA-SFM* Committé Antibiogramme – Société Française de Microbiologie (CA-SFM), *EUCAST* European Committee on Antimicrobial Susceptibility Testing, *NCCLS* National Committee for Clinical Laboratory Standards, *CLSI* Clinical and Laboratory Standards Institute, *API* Analytical profile index, *NAAT* Nucleic acid amplification tests, *MALDI-ToF* Matrix Assisted Laser Desorption/Ionization-Time of Flight, *ISO* International Organization for Standardization, *EQA* External Quality Assurance; ¥Polymerase Chain Reaction

^a^Studies might have used more than one method and they were counted more than once

^b^Two studies mentioned a non-specific accreditation and National Accreditation; Hospital acquired infection (HAI) was defined as a new clinical infections in patients who had been admitted for ≥48 h in a hospital setting. Community acquired infection was defined as infection occurring in the community or within 48 h of hospital admission

### Microbial resistance patterns

The most commonly reported bacterium was *Escherichia coli (*87/144, 60.4%) with the most frequent susceptibility data for gentamicin (77/144, 53.5%), ciprofloxacin (71/144, 49.3%) and sulfamethoxazole/trimethoprim (68/144, 47.2%) (Table 2). In total, 13 Gram negative opportunistic pathogens were tested against 37 antibiotics (Table 3, Additional file 2). Overall resistance to commonly used drugs [8, 9, 12], like amoxicillin (MR 72.9%, IQR9.1%–87.3%) and trimethoprim/sulfamethoxazole (MR 75.0%, IQR 49.5%–92.3%) was high. Low to moderate resistance was found to gentamicin (MR 22.1%, IQR 2.0%–45.0%), ciprofloxacin (MR 16.7%, IQR 0%–38.5%) and ceftriaxone (MR 17.2%, IQR 0%-45.0%). Gentamicin resistance in *Klebsiella* spp., which is naturally resistant to ampicillin, was reported in 1031/2715, 38.0% of tested isolates. Resistance to either ceftriaxone or cefotaxime, which is suggestive of extended–spectrum beta lactamase (ESBL) production was reported in 593/2963, 20.0% and 1051/5395, 19.5% of *E. coli* as well as 545/1594, 34.2% and 560/1199, 46.7% of the *K. pneumoniae* isolates, respectively. Imipenem, a drug rarely available in rural Africa, was investigated in 21/144 (14.6%) of the studies and showed the lowest overall MR (MR 3.0%, IQR 0%- 26.6%) (Table 3). Of the *Acinetobacter* isolates reported in three studies, 32.3% (10/31) were resistant to meropenem. Ciprofloxacin resistance in S. Typhi was reported rarely (MR 0%, IQR 0%–11.7%) while ampicillin resistance in *Haemophilus influenza* was high (MR 100%, IQR76.6%–100%). There was no documented ceftriaxone resistance in *N. gonorrhoeae* but ciprofloxacin resistance was reported in 37.5% (IQR 30.7%–83.9%) of the isolates.

**Table 2:** Bacteria reported as number of studies (% of all of studies) in different clinical syndromes

Bacteria	No. Studies	HAI N (%)	BSI N (%)	UTI N (%)	AGE N (%)	WI N (%)	Carriage N (%)
*S. aureus*	79	17 (21.5)	17 (21.5)	NS	NS	22 (27.8)	10 (12.7)
*S. pneumoniae*	24	NA	8 (33.3)	1 (4.2)	NS	NS	4 (16.7)
*E. coli*	87	15 (17.2)	17 (19.5)	17 (19.5)	7 (8)	NS	2 (2.3)
*Enterobacter* spp.	36	3 (8.3)	8 (22.2)	8 (22.2)	1 (2.8)	NS	NA
*Haemophilus influenza*	7	NA	NA	NS	NS	NS	NA
CoNS^a^	27	9 (33.3)	6 (22.2)	5 (18.5)	NS	9 (33.3)	NA
*Citrobacter* spp.	19	2 (10.5)	5 (26.3)	7 (36.8)	NS	NS	NA
S*.* Typhi	28	2 (7.1)	13 (46.4)	1 (3.6)	6 (21.4)	NS	1 (3.6)
*Pseudomonas aeruginosa*	60	17 (28.3)	11 (18.3)	8 (13.3)	NS	20 (33.3)	2 (3.3)
*Serratia* spp.	7	1 (14.3)	2 (28.6)	NA	NS	NS	1 (14.3)
*Klebsiella pneumoniae*	75	15 (20)	21 (28)	15 (20)	NS	16 (21.3)	2 (2.7)
*Proteus* spp.	46	10 (21.7)	8 (17.4)	10 (21.7)	NS	NS	1 (2.2)
*Acinetobacter* spp.	17	5 (29.4)	7 (41.2)	1 (5.9)	NS	4 (23.5)	NA
*Neisseria meningitidis*	1	NA	NA	NS	NS	NS	NA
*Shigella* spp.	8	NA	NA	NA	6 (75)	NS	NA
*Campylobacter* spp.	3	NA	NA	1 (33.3)	3 (100)	NS	NA
*Group A Streptococcus*	18	1 (5.6)	6 (33.3)	NS	NS	3 (16.7)	2 (11.1)
*Group B Streptococcus*	2	NA	NA	NS	NS	NA	NA
*Listeria monocytogenes*	2	NA	1 (50)	1 (50)	NS	NA	NA
Non-Typhoidal *Salmonella* spp.	11	NA	9 (81.8)	NS	NA	NS	NA
*Neisseria gonorrhoeae*	11	NS	NA	2 (18.2)	NS	NS	NA
*Moraxella* spp.	2	NS	NA	NS	NS	NS	NA
*Streptococcus* spp.	3	NA	NA	1 (33.3)	NS	1 (33.3)	1 (33.3)

*HAI* Hospital Acquired Infection, *UTI* Urinary Tract Infection, *AGE* Acute Gastroenteritis, *WI* Wound Infection, *BSI* Bloodstream Infections, *NS* Not significant since the bacterium is an unlikely cause of the infection etiology [180], *NA* Not applicable because data were not available for the specific combination

^a^CoNS: *Coagulase Negative Staphylococcus* spp.

**Table 3:** Median resistance with interquartile range of selected Gram negative bacteria to all tested antibiotics

Drugs	*Acinetobacter* spp. *(N Isolates)* *Median (IQR)*	*Citrobacter* spp. *(N Isolates)* *Median (IQR)*	*Escherichia coli* *(N Isolates)* *Median (IQR)*	*Haemophilus influenzae* *(N Isolates)* *Median (IQR)*	*Klebsiella pneumoniae* *(N Isolates)* *Median (IQR)*	*Neisseria gonorrhoeae* *(N Isolates)* *Median (IQR)*	Non-Typhoidal *Salmonella* spp. *(N Isolates)* *Median (IQR)*	*Proteus* spp. *(N Isolates)* *Median (IQR)*	*Pseudomonas aeruginosa* *(N Isolates)* *Median (IQR)*	*Salmonella enteric serovar* Typhi *(N Isolates)* *Median (IQR)*	*Shigella* spp. *(N Isolates)* *Median (IQR)*
Amikacin	(240) 32.7(0–63.6)	(29) 5.8(0–15.4)	(5422) 0.2(0–24.5)	*NC	(1458) 12.6(1.6–37.7)	NC	NC	(251) 16.7(0–67.6)	(476) (6.5–50)	(433) 4.9(0.2–15.5)	NC
Amoxicillin	**NC**	**(71)** **78.5(73.4–95.8)**	**(5500)** **88.1(81.4–92.6)**	**NC**	**(524)** **100(86.8–100)**	**NC**	**NC**	**(409)** **82(48.8–91.4)**	**(230)** **84.8(19.2–98.2)**	**(150)** **69.2(44.0–77.3)**	**NC**
Ampicillin	**(188)** **100(92.5–100)**	**(59)** **100(66.1–100)**	**(2951)** **86.7(69.2–96.7)**	**(18)** **100(76.6–100)**	**(1259)** **100(93.4–100)**	**NC**	**NC**	**(685)** **42.3(28.3–84.7)**	**(820)** **100(94.5–100)**	**(380)** **57.8(21.2–75)**	**(110)** **51.1(45.0–61.6)**
Amoxicillin and Clavulanic Acid	**(56)** **94.1(15–100)**	**(53)** **34.8(0–100)**	**(6764)** **43.5(30.8–61.9)**	**(35)** **22.2(0–39.5)**	**(2043)** **55.4(35.7–73.2)**	**NC**	**(357)** **0(0–53.4)**	**(730)** **21.2(0–56.3)**	**(852)** **50(3.1–91.9)**	**(558)** **6.1(0.6–15.1)**	**NC**
Azithromycin	NC	NC	NC	NC	NC	(199) 4.2(2.2–33.3)	(1120) 0(0–5.2)	NC	NC	(197) 0(0–0.5)	NC
Cefotaxime	(205) 90.9(41.7–100)	(58) 29.2(9.1–72.8)	(5173) 26.8(8.3–64.5)	NC	(1199) 50(32.5–76.4)	(96) 0(0–3.4)	(1099) 2.1(0–11.7)	(324) 20.8(0.3–57.6)	(288) 88.5(18.3–100)	(509) 0(0–1.2)	NC
Ceftazidime	(260) 81.5(36.1–90.8)	(60) 45(9.8–78.6)	(2773) 19.5(10.0–55.8)	NC	(1412) 46.5(12.7–62.9)	NC	NC	(463) 30.9(4.7–39.9)	(1216) 29(25–46.9)	(512) 1.2(0–39.0)	(354) 0(0–22.2)
Ceftriaxone	(69) 74.2(38.3–100)	(116) 25.3(0–77.2)	(2800) 31.5(6.9–47.7)	(88) 20.4(0–57.1)	(1547) 47.3(25–62.8)	(584) 0	(807) 9(0.2–41.8)	(755) 17(1.1–35.3)	(915) 30.6(4.5–78.5)	(150) 0(0–19.5)	NC
Cefuroxime	(31) 75(45.8–100)	NC	(3925) 30(19.7–51.2)	NC	(947) 51.9(35.8–83.2)	NC	NC	(124) 30.7(1.1–59.2)	NC	NC	NC
Cefoxitin	NC	NC	(535) 8.3(2.9–44.1)	NC	NC	NC	NC	NC	NC	NC	NC
Cefepime	(79) 50(0–75.2)	NC	(957) 21.8(5.8–42.5)	NC	NC	NC	NC	NC	NC	NC	NC
Cefalotin	NC	NC	(515) 56.9(23.5–63.5)	NC	(154) 55.7(42.4–76)	NC	NC	NC	NC	(145) 6.8(2.4–35.3)	(201) 3.8(0–8.5)
Chloramphenicol	**(47)** **100(60.4–100)**	**(88)** **70.8(18.8–91.7)**	**(2963)** **40.9(11.3–58.0)**	**(56)** **28.6(10.9–100)**	**(1046)** **62.8(48.9–91.5)**	**NC**	**(1982)** **60.5(34.9–88)**	**(538)** **50(14.3–76.4)**	**(691)** **67(33.8–91.7)**	**(348)** **43.1(25–60)**	**(65)** **11.1(9.1–27.8)**
Ciprofloxacin	**(47)** **29.9(12.1–74.9)**	**(147)** **10(0–31.2)**	**(7877)** **23.2(7.7–35.6)**	**(28)** **14.6(3.1–79.2)**	**(2473)** **24.3(7.8–35.4)**	**(658)** **37.5(30.7–83.9)**	**(1147)** **0.4(0–15.7)**	**(924)** **7.2(0–27.4)**	**(1083)** **16.1(2–38.4)**	**(562)** **0(0–11.7)**	**(386)** **0(0–5.4)**
Trimethoprim/ Sulfamethoxazole	**(133)** **80.4(54.5–100)**	**(122)** **85.7(64.3–100)**	**(7493)** **80.7(69.8–85.6)**	**(54)** **83.8(0–100)**	**(2093)** **67.8(60.5–93.0)**	**(72)** **92.6(25–100)**	**(1981)** **60.5(40–88.8)**	**(833)** **61.3(39.1–78.7)**	**(912)** **92(75.7–100)**	**(1001)** **52.8(29.4–72.9)**	**(392)** **83.3(39.9–88)**
Doxycycline	NC	NC	(302) 54.5(12.8–72.3)	NC	(531) 67.8(60.5–93.0)	NC	NC	NC	NC	NC	NC
Erythromycin	NC	NC	(675) 81.6(29.9–86.5)	NC	(444) 58.9(39.8–82.6)	NC	NC	NC	(501) 77.3(40.8–100)	NC	NC
Gentamicin	(288) 61.1(25–86)	(143) 37.5(0–48.4)	(8282) 29.8(8.4–45.3)	(35) 59.4(8.9–100)	(2691) 41.7(24.1–66.7)	(88) 28.6(0–55.2)	(1657) 11.9(4.9–37)	(1027) 13.5(0–34)	(1554) 29.4(17.7–44.5)	(801) 2.3(0–21.7)	(365) 5.9(0.2–23.2)
Imipenem	(230) 2(0–23)	NC	(1613) 0.2(0–5.5)	NC	(1002) 0(0–6.3)	NC	NC	(21) 10.7(0–67.8)	(486) 6(0–51.8)	NC	NC
Levofloxacin	NC	NC	(751) 19.2(8.7–47.6)	NC	(246) 15.6(8.6–62.7)	NC	NC	(138) 42.7(4.7–91.7)	(77) 25.8(16.5–36.5)	NC	NC
Meropenem	(24) 40(0–44.4)	NC	(3402) 0(0–5.7)	NC	(711) 3.6(0–11.7)	NC	NC	(154) 1(0–1.6)	(138) 15.8(0–32)	NC	NC
Nalidixic Acid	NC	(39) 42.9(41.7–62.2)	(2960) 36(12.7–53.8)	NC	(507) 35.2(14.8–53)	NC	(1007) 10.6(4.4–19.6)	(166) 59.3(34–93.8)	(101) 78.4(54.2–90.8)	(537) 5.7(0.6–19.3)	(367) 0.6(0–17.3)
Nitrofurantoin	(27) 60(0–75)	(52) 40.6(25–52.3)	(5087) 14(4.5–25.1)	NC	(957) 24.0(13–54.7)	NC	NC	(257) 56.3(33.1–84.2)	(151) 76.5(65.9–80)	NC	NC
Norfloxacin	(39) 34.5(6.3–86)	(89) 41(0–35.6)	(876) 25.6(15.0–46.3)	NC	(518) 31.2(5.6–43.6)	NC	NC	(303) 0(0–3.3)	(578) 17(0–75)	NC	NC
Ofloxacin	NC	NC	(1294) 30.4(9.8–47.9)	NC	(733) 17(0–23.4)	NC	(1007) 73.2(43.7–90.5)	(322) 29(0–67)	NC	NC	NC
Oxacillin	NC	NC	(411) 91.5(22.2–98.5)	NC	NC	NC	NC	(128) 46.7(11.9–82.9)	(474) 100(33.3–100)	NC	NC
Penicillin	NC	NC	(43) 62(52.9–90.6)	NC	NC	(564) 75(52.4–100)	NC	NC	NC	NC	NC
Piperacillin	(78) 50(41.7–75)	NC	(132) 58.4(35.1–95.0)	NC	NC	NC	NC	NC	NC	NC	NC
Piperacillin/ Tazobactam	(83) 33.6(12.7–80.1)	NC	(235) 21(11.1–30.6)	NC	NC	NC	NC	NC	(95) 14.6(2.6–45.8)	NC	NC
Tetracycline	(90) 73(62.2–100)	(72) 83.3(45–100)	(2896) 76.2(72.6–87.9)	(43) 25(11.1–25)	(744) 68(58.7–89.4)	(544) 91.7(63.6–100)	(1532) 37.1(22.4–66.3)	(455) 88.2(56.4–95.8)	(352) 100(81.8–100)	(309) 43(7.5–72.3)	(92) 54.5(22.2–71.1)
Tobramycin	NC	NC	(677) 32(12.3–43.2)	NC	(207) 55(13.0–67.8)	NC	NC	NC	NC	NC	NC

Antibiotics which are routinely available and recommended as first or second line antibiotics according to IMNCI and IMAI empiric guidelines are highlighted in bold [8, 9]

NA Not Applicable: Bacterium is naturally resistant to agentAntibiotic is not recommended for treatment of this bacterium since resistance is likely to be present

* NC Not Calculated: MR and IQR not calculated because of a small number of studies for the specific combination

In the Gram positive group, Coagulase Negative *Staphylococcus* spp*.* (CoNS), *S. aureus*, *Streptococcus pneumoniae* and Group A streptococcus were the most commonly investigated bacteria (Table 4). Methicillin resistant *S. aureus* (MRSA) was reported in 7/79 (8.9%) of the studies, however as cefoxitin is typically used to screen for MRSA, the MRSA rate is likely underestimated [15]. Erythromycin resistance in *S. aureus* was found in 33.9% (IQR 13%–46.4%). Methods used to assess penicillin susceptibility in *S. pneumoniae* varied: median oxacillin resistance was reported in 40.7% (IQR 0%–55.7%) compared to 26.7% (IQR 8.4%–33.6%) for penicillin and 22.5% (IQR 20%–32.5%) for amoxicillin.Vancomycin showed the lowest resistance pattern for all the tested Gram positive bacteria (Table 4).

**Table 4:** Median resistance with interquartile range of selected Gram positive bacteria to all tested antibiotics^a^

Antibiotics	CoNS *(N Isolates)* *Median (IQR)*	Group A *Streptococcus* *(N Isolates)* *Median (IQR)*	*S. aureus* *(N Isolates)* *Median (IQR)*	*S. pneumoniae* *(N Isolates)* *Median (IQR)*
Amikacin	NC	NC	(472) 3 (0–8)	NC
Amoxicillin	**NC**	**(96)** **14.3 (0–100)**	**(1090)** **78.6 (62.5–91.7)**	**(229)** **22.5 (20–32.5)**
Ampicillin	**(522)** **78.9 (41.3–97)**	**(108)** **7.9 (0–41)**	**(2355)** **85.7 (78.4–93.8)**	**(436)** **20 (6.5–37.8)**
Amoxicillin and Clavulanic Acid	**(228)** **31.8 (5–50.7)**	**NC**	**(2413)** **23.5 (11.4–40.2)**	**(331)** **17.4 (3.4–70)**
Cefotaxime	(142) 51.2 (28.7–79.3)	NC	(403) 28.1 (2.7–39.5)	(156) 3.6 (0–12.8)
Cefoxitin	NC	NC	(1717) 10.4 (4.6–33.8)	NC
Ceftazidime	NC	NC	(1141) 50 (25.8–54.2)	NC
Ceftriaxone	(366) 43.9 (9.9–65.6)	(155) 0 (0–16.2)	(2206) 38 (11–47)	(690) 2.2 (0–8.8)
Cefuroxime	(58) 60 (48–68.6)	NC	(686) 49.2 (32.5–62.6)	(267) 6.6 (0.6–19.8)
Chloramphenicol	**(445)** **51.2 (37.4–77.3)**	**(144)** **26 (4.6–62.5)**	**(2413)** **24.1 (1.3–54.3)**	**(776)** **20.7 (0.9–24.9)**
Ciprofloxacin	**(568)** **42.9 (22.9–54.5)**	**(90)** **18.9 (10.7–100)**	**(2678)** **21.1 (8.5–33.7)**	**(452)** **13.5 (4.1–70.1)**
Clindamycin	NC	(113) 0 (0–7.5)	(1400) 11.7 (0–47.4)	(185) 6.1 (0–13.2)
Sulfamethoxazole/Trimethoprim	**(496)** **66.7 (59.3–85.3)**	**(163)** **33.9 (0–64.7)**	**(3948)** **66.1 (42.8–84.3)**	**(976)** **90.4 (71.4–98.6)**
Doxycycline	**NC**	**NC**	**(777)** **55.5 (40–79.9)**	**NC**
Erythromycin	**(476)** **48 (38.5–76)**	**(197)** **10.4 (3.8–55.2)**	**(3796)** **33.9 (13–46.4)**	**(785)** **11.5 (2.7–21.5)**
Gentamicin	**(748)** **26.5 (22.2–42.1)**	**(83)** **24.3 (0–40.8)**	**(4422)** **18.7 (1.5–32.1)**	**(322)** **24.5 (15.4–88.5)**
Imipenem	NC	NC	(126) 8 (1.9–28.9)	NC
Levofloxacin	NC	NC	(1148) 5.9 (0.4–16.1)	NC
Nalidixic Acid	NC	NC	(292) 73.2 (67.5–82)	NC
Nitrofurantoin	(89) 14.7 (0–72.2)	NC	(294) 21.8 (4.5–38.2)	NC
Norfloxacin	NC	(37) 24.3 (0–0)	(1165) 30 (4.8–33)	NC
Ofloxacin	NC	NC	(1214) 26.4 (6.4–45.1)	NC
Oxacillin	**(467)** **48 (24.5–67.7)**	**(29)** **0 (0–75)**	**(2665)** **34.5 (12.6–68.2)**	**(270)** **40.7 (0–55.7)**
Penicillin	**(275)** **67.4 (31.6–98)**	**(70)** **0 (0–16.2)**	**(1991)** **90.4 (80.8–96.4)**	**(1125)** **26.7 (8.4–33.6)**
Tetracycline	**(603)** **56.6 (41.4–77.2)**	**(37)** **25 (0–63.5)**	**(3297)** **44.8 (27.7–58.7)**	**(703)** **34.4 (18.8–65.4)**
Vancomycin	(384) 1.6 (0–34.6)	NC	(2079) 2.8 (0–10.2)	(274) 0 (0–10.2)

Antibiotics routinely available and recommended as first or second line antibiotics according to IMNCI and IMAI empiric guidelines are highlighted in bold [8, 9]

NA Not Applicable: Bacterium is naturally resistant to agent

NC Not Calculated: MR and IQR not calculated because of a small number of studies for the specific combination

^a^The four most frequently isolated Gram positive bacteria were included in this table

In the reviewed literature*, E. coli* was commonly isolated from patients with BSI (17/87, 19.5%), UTI (17/87, 19.5%) and wound infection (16/87, 18.4%). *Citrobacter* spp. were reported most commonly among patients with UTIs (7/19; 36.8%), followed by BSI (5/19, 26.3%). Two (2/19, 10.6%) [16, 17] of the studies which reported on *Citrobacter* spp. were from HAI; five (5/19, 26.3%) from community acquired infections of which three (3/5) came from pregnant women at antenatal care follow up [18–20] and two (2/5) had unknown risk factors [21, 22]. *S*. Typhi was most frequently reported from patients with BSI (13/28, 46.4%) followed by patients with diarrhea (6/28, 21.4%). It was also more commonly reported in children below 18 years (12/28, 48.9%) than in adults (2/28, 7.1%) (Table 2).

In the current review, the susceptibility results were taken at face value, however there were inconsistencies casting the credibility of several results in doubt. For example a study reporting more than 80% of *Proteus* isolates (*n* = 6) as resistant to imipenem also reported that 80% of them were susceptible to ceftriaxone and 50% were susceptible to vancomycin, a combination that is highly unusual [23]. There were other unverified reports of highly unusual resistance patterns from some centers, such as penicillin resistant *S. pyogenes* [24, 25] and vancomycin-resistant *S. aureus* [26, 27].

### Regional antibiotic resistance patterns

**Fig. 3:**
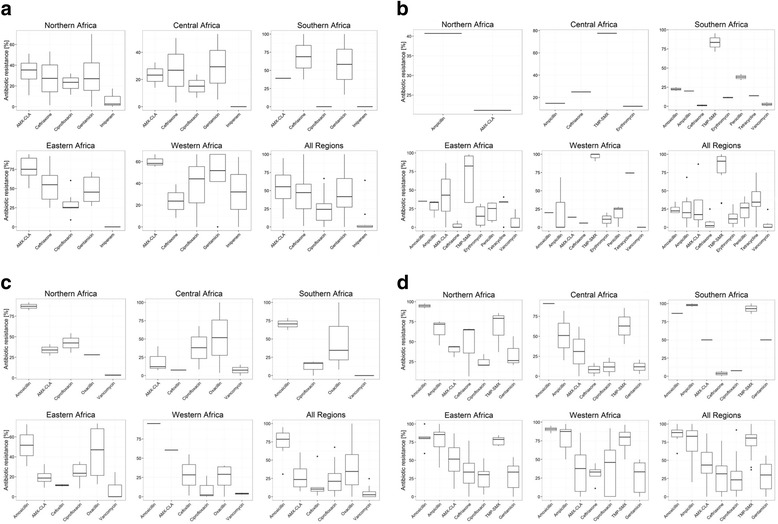
Resistance of selected pathogens to commonly prescribed antibiotics in different regions of Africa. The boxplots in the figure represent the median and interquartile range of resistance reported if at least three studies reported on the combination. Resistance to amoxicillin-clavulanic acid (AMX-CLA), ampicillin, amoxicillin, penicillin, oxacillin, trimethoprim-sulfamethoxazole (TMP-SXT), gentamicin, ceftriaxone, cefoxitin, ciprofloxacin, erythromycin, tetracycline, vancomycin and imipenem were plotted. Antibiotics with no data points in the specific regions are omitted from the plots.Resistance patterns reported using broth dilution minimum inhibitory concentration (MIC), E-test® or VITEK® were included if prevalence could be calculated and were combined with resistance data reported using disk diffusion as this was the main method used. Intermediate susceptible strains were categorized as resistant to simplify the analysis. [13]MR estimates were not calculated if only one or two studies reported on the specific bacterium-antibiotic combination. **a**: Resistance of Klebsiella spp. to commonly prescribed antibiotics in different regions of Africa. **b**: Resistance of S. pneumoniae to commonly prescribed antibiotics in different regions of Africa. **c**: Resistance of S. aureus to commonly prescribed antibiotics in different regions of Africa. **d**: Resistance of E. coli to commonly prescribed antibiotics in different regions of Africa

Generally, a lower level of resistance of *S. aureus, Klebsiella* spp.*, E. coli* and *S. pneumoniae* to carbapenems and fluoroquinolones was observed in all the regions as compared to the other antibiotic-bacterium combinations. However, *Klebsiella* spp. resistance to ciprofloxacin in West Africa was observed to be higher than in other regions. Resistance to the trimethoprim (MR: 33.9%–100%), ampicillin (MR: 7.9%–100%) and penicillin (MR: 0%–75%) was generally high in all regions (Fig. 3).

## Discussion

The rise and spread of AMR threatens the effective control and treatment of various bacterial diseases worldwide [1, 2]. The achievements gained in reducing mortality and morbidity through early use of antibiotics based on empiric guidelines are in serious jeopardy if appropriate actions are not taken to control AMR [28, 29]. Availability of routine and research data on pathogen susceptibilities is an important step towards designing targeted strategies to tackle the global AMR crisis. The current review describes recently (2013–2016) published data on antibiotic drug susceptibility from Africa.

The lack of consistency in the measurement and reporting of susceptibility data makes it difficult to compare findings among different countries and laboratories, sometimes even within one country [30, 31]. To address this issue, high income countries have implemented harmonization efforts. For example, laboratories in Europe are encouraged to use the European Committee on Antimicrobial Susceptibility Testing (EUCAST) standard over the Clinical and Laboratory Standards Institute (CSLI) guidelines [32]. Furthermore, in an effort to enable coherent data synthesis and reporting in January 2016 the British Society for Antimicrobial Chemotherapy (BSAC) actively promoted the EUCAST methods in favour of the current BSAC methodology [33]. Given the findings of our review, similar harmonization efforts are urgently needed in Africa. Standardizing AMR methods and interpretation guidelines could allow for better comparability of results and improved resistance tracking. Furthermore, improved access to reference laboratories and EQA schemes are needed augmenting the current WHO initiative to scale up the global antimicrobial surveillance system (GLASS) based on country specific priority pathogens [34]. Currently, in the absence of a uniform laboratory methodology the GLASS goals will be very difficult to meet.

Comparing our findings with previous reviews in the region like Leopold et al. (2014), overall, we identify a similarly high report of resistance to commonly used antibiotics [13]. The same review reported a high level of resistance of Enterobacteriaceae to ampicillin and co-trimoxazole which is in agreement with our findings. Similarly, resistance to co-trimoxazole and tetracycline by *S. pneumoniae* was reported to be high. However, discrepancies were observed in various antibiotic/bacterium combinations. For instance, our finding of resistance to chloramphenicol in *Salmonella* Typhi isolates is lower than that previously reported [13]. Resistance to oxacillin by *S. aureus* was also much higher in the current review than the review by Leopold et al. [13]. The observed differences between data published in 2014 and the current work could indicate a rising pattern in AMR in certain pathogens. However it could also be because of the differences in AMR testing methodologies underlining the need for harmonization of laboratory methods in the region.

A notable finding of this review was the high resistance rate of common causes of UTI to common first line regimes like amoxicillin and sulfamethoxazole/trimethoprim. In the presence of a failing treatment, patients with UTIs are at increased risk of developing renal damage and future risks of renal insufficiency or hypertension [35]. Similarly, given the resistance profiles in the current review, neonatal sepsis or BSI caused by *E. coli*, *K. pneumoniae* and *S. aureus* are not being effectively treated by first line drugs like ampicillin, aminoglycosides and cephalosporins, which will result in increased mortality in patients with life-threatening infections. The high levels of resistance to amoxicillin and penicillin in *S. pneumoniae* and *H. influenzae* are also concerning given that pneumonia is a leading cause of death in children [36, 37]. Reported MRSA rates were variable and doubts remain about reliability of identification at all sites which are confirmed by the findings of one Kenyan study which found that MRSA rates dropped dramatically after switching to an automated identification method [38].

Compared to reports from Asia, quinolone resistance in *S*. Typhi was rare and, reassuringly, there were no reports of ceftriaxone resistance in *N. gonorrhoeae* [39]. Less commonly prescribed antibiotics like imipenem and vancomycin also showed low level resistance and they should be preserved as alternative drugs in severe infections. Most of the imipenem resistance was described in isolates of *P. aeruginosa* which has been reported from other centers [33]. Oxacillin resistance should predict penicillin resistance in *S. pneumoniae* reliably [40, 41], however in the current review resistance to oxacillin was much higher than to penicillin, possibly because of the differences in the number of isolates tested for both antibiotics and the use of different cut-offs for meningitis and non-meningitis strains .

The results also yielded data on the susceptibility of less commonly described bacteria like *Acinetobacter* spp. and *Citrobacter* spp. *Acinetobacter* spp. are especially important given their importance in clinical infections and the reported rising trend of resistance, further they have been included in the priority pathogens for global surveillance based on the GLASS initiative [42]. *Citrobacter* spp. were reported from hospital and community settings, including in pregnant mothers and can cause UTI which puts pregnant women at risk of preterm labor. Our results would suggest that current frontline treatments are ineffective against most common uro-pathogens.

The limitations of the current review include the exclusion of non-English language reports, as articles from French speaking African countries might have been missed, biasing this review. The representativeness of the data is hard to assess as it is possible that the absence of resistance is not routinely reported and focus is given to reports of resistance. There were very few reports from South Africa, which has a better functioning health system than neighboring countries and better national AMR surveillance. These data were not accessible by our search and therefore larger AMR trends might have been missed. A further limitation is combining AMR results from different patient groups across different countries to compare the data. This approach might have leveled out peaks of resistance in different settings. However, given the observed trends, we believe that the resolution of the obtained data was sufficient to show general developments. Moreover, since case reports were not excluded with intention of capturing as much data as possible in the current review, the findings from the three case (3/144, 2.1% of the studies) might bias the resistance testing results as case reports tend to report on specific multi-drug resistant pathogens. Finally, resistance data obtained with different laboratory methodologies were combined for the purposes of this review. However, as the majority of studies used the disk diffusion method and CLSI guidelines, the impact of the variation in AMR methodology on the validity of the final results is thought to be minimal.

## Conclusion

In summary, our review highlights three important findings: first, more than a third of the countries on the continent did not have recent AMR data published in the public domain and only a few of those were surveillance data. Second, a high level of drug resistance exists to commonly prescribed antibiotics in the African continent. Third, the standardization and quality of the microbiological identification and susceptibility testing methods needs to be improved to allow national and international organizations to monitor the extent of the AMR problem. All of the identified areas of concern need urgent attention by the global health community in order to halt the public health threat associated with spreading AMR.

## Additional files

Additional file 1: Detailed Methododologies (DOCX 24 kb)

Additional file 2: Proportion of resistance of bacterial isolates to tested antibiotics by region and country (DOCX 263 kb)
