# Supplementary material for: Antimicrobial resistance in Africa: a systematic review
Source: BMC Infect Dis. 2017 Sep 11;17:616. doi: 10.1186/s12879-017-2713-1 (PMC5594539; doi:10.1186/s12879-017-2713-1)
Supplement: Supplementary file 1 — Detailed Methododologies (DOCX 24 kb) [file 12879_2017_2713_MOESM1_ESM.docx]

**Additional file 1 Detailed Methododologies**

**Search strategy**

Strategy from January 1 2013 to January 31 2016) from the different databases.

PubMed (February 03, 2016) (559):

"Drug Resistance, Bacterial"[Mesh] or “Microbial Sensitivity Tests”[Mesh] or (antibiotic resistan*[tiab]) or (antibacterial resistan*[tiab]) or (antibacterial drug resistan*[tiab]) or (bacterial drug resistan*[tiab]) or (microbial drug resistan*[tiab]) or (microbial resistan*[tiab]) or (antimicrobial resistan*[tiab]) or (antibiotic drug resistan*[tiab]) or (bacterial resistan*[tiab]) or (antibiotics resistan*[tiab]) or (bacterial surveillan*[tiab]) or (antibiotic surveillan*[tiab]) or (antibacterial surveillan*[tiab]) or (antimicrobial surveillan*[tiab] or (antibiotic susceptibility [tiab] ) OR (Virulen*[tiab])) AND ((Africa*[tiab]) OR "Comoros"[Mesh] OR "Djibouti"[Mesh] OR "Madagascar"[Mesh] OR "Malawi"[Mesh] OR "Seychelles"[Mesh] OR "Cameroon"[Mesh] OR "Central African Republic"[Mesh] OR "Chad"[Mesh] OR "Congo"[Mesh] OR "Equatorial Guinea"[Mesh] OR "Atlantic Islands"[Mesh] OR (Gabon*[tiab]) OR "Morocco"[Mesh] OR "South Sudan"[Mesh] OR "Sudan"[Mesh] OR "Botswana"[Mesh] OR "Lesotho"[Mesh] OR "Swaziland"[Mesh] OR "Benin"[Mesh] OR "Burkina Faso"[Mesh] OR "Cape Verde"[Mesh] OR "Ghana"[Mesh] OR "Guinea"[Mesh] OR "Guinea-Bissau"[Mesh] OR "Mauritania"[Mesh] OR "Niger"[Mesh] OR "Senegal"[Mesh] OR "Sierra Leone"[Mesh] OR "Togo"[Mesh] OR (Burundi*[tiab]) or (eritrea*[tiab]) or (ethiopia*[tiab]) or (kenya*[tiab]) or (mozambique*[tiab]) or (rwanda*[tiab]) or (somalia*[tiab]) or (tanzania*[tiab]) or (uganda*[tiab]) or (zambia*[tiab]) or (zimbabwe*[tiab]) or (angola*[tiab]) or (algeria*[tiab]) or (egypt*[tiab]) or (tunisia*[tiab]) or (namibia*[tiab]) or (south africa*[tiab]) or (gambia*[tiab]) or (liberia*[tiab]) or (mali*[tiab]) or (Nigeria*[tiab])) NOT (candid*[tiab]) NOT "Antitubercular Agents"[Mesh] NOT "Antiviral Agents"[Mesh] NOT "Antimalarials"[Mesh] NOT "Vaccines"[Mesh] NOT "Neoplasms"[Mesh] NOT "Anti-Retroviral Agents"[Mesh] NOT "Antiparasitic Agents"[Mesh] NOT (fung*[tiab]) AND Humans[Mesh] AND English[lang]

## EMBASE SEARCH (Februrary 03, 2016)

## Search Queries

| No. | Query | Results | Date |
| --- | --- | --- | --- |
| #51 | (((((((((((((((((('africa'/exp OR comoros OR djibouti OR madagascar OR malawi OR seychelles OR cameroon OR 'central african republic' OR chad OR congo OR 'equatorial guinea' OR 'atlantic islands' OR gabon OR morocco OR 'south sudan' OR sudan OR botswana OR lesotho OR swaziland OR benin OR 'burkina faso' OR 'cape verde' OR ghana OR guinea OR 'guinea-bissau' OR mauritania OR niger OR senegal OR 'sierra leone' OR togo OR 'burundi*' OR 'eritrea*' OR 'ethiopia*' OR 'kenya*' OR 'mozambique*' OR 'rwanda*' OR 'somalia*' OR 'tanzania*' OR 'uganda*' OR 'zambia*' OR 'zimbabwe*' OR 'angola*' OR 'algeria*' OR 'egypt*' OR 'tunisia*' OR 'namibia*' OR 'south africa*' OR 'gambia*' OR 'liberia*' OR 'mali*' OR 'nigeria*') AND ('anti-bacterial resistance' OR 'antibiotic resistan*' OR 'antimicrobial resistan*' OR 'drug resistan*' OR 'multi-drug resistan*' OR 'multidrug resistan*' OR 'multiple drug resistan*' OR 'antibiotic susceptib*' OR 'antimicrobial* susceptib*' OR 'drug* susceptib*' OR 'multi-drug susceptib*' OR 'multidrug susceptib*' OR 'multiple-drug susceptib*' OR 'multiple drug* susceptib*')) NOT 'antimalarial'/exp) NOT 'antituberculosis agent'/exp) NOT 'virus'/exp) NOT 'fungus'/exp) NOT 'malaria'/exp) NOT 'tuberculosis'/exp) NOT 'hiv'/exp) NOT 'cancer'/exp) NOT 'vaccine'/exp) NOT 'drug trial'/exp) NOT 'parasite'/exp) NOT cancer) NOT molecular AND [1-1-2013]/sd NOT [3-2-2016]/sd) NOT 'giardia'/exp AND ([embase]/lim OR [embase classic]/lim) AND [humans]/lim AND [english]/lim AND [abstracts]/lim) NOT 'helminth'/exp) NOT 'human immunodeficiency virus'/exp) NOT 'protozoa'/exp | 1093 | 3 Feb 2016 |
| #50 | (((((((((((((((((('africa'/exp OR comoros OR djibouti OR madagascar OR malawi OR seychelles OR cameroon OR 'central african republic' OR chad OR congo OR 'equatorial guinea' OR 'atlantic islands' OR gabon OR morocco OR 'south sudan' OR sudan OR botswana OR lesotho OR swaziland OR benin OR 'burkina faso' OR 'cape verde' OR ghana OR guinea OR 'guinea-bissau' OR mauritania OR niger OR senegal OR 'sierra leone' OR togo OR 'burundi*' OR 'eritrea*' OR 'ethiopia*' OR 'kenya*' OR 'mozambique*' OR 'rwanda*' OR 'somalia*' OR 'tanzania*' OR 'uganda*' OR 'zambia*' OR 'zimbabwe*' OR 'angola*' OR 'algeria*' OR 'egypt*' OR 'tunisia*' OR 'namibia*' OR 'south africa*' OR 'gambia*' OR 'liberia*' OR 'mali*' OR 'nigeria*') AND ('anti-bacterial resistance' OR 'antibiotic resistan*' OR 'antimicrobial resistan*' OR 'drug resistan*' OR 'multi-drug resistan*' OR 'multidrug resistan*' OR 'multiple drug resistan*' OR 'antibiotic susceptib*' OR 'antimicrobial* susceptib*' OR 'drug* susceptib*' OR 'multi-drug susceptib*' OR 'multidrug susceptib*' OR 'multiple-drug susceptib*' OR 'multiple drug* susceptib*')) NOT 'antimalarial'/exp) NOT 'antituberculosis agent'/exp) NOT 'virus'/exp) NOT 'fungus'/exp) NOT 'malaria'/exp) NOT 'tuberculosis'/exp) NOT 'hiv'/exp) NOT 'cancer'/exp) NOT 'vaccine'/exp) NOT 'drug trial'/exp) NOT 'parasite'/exp) NOT cancer) NOT molecular AND [1-1-2013]/sd NOT [3-2-2016]/sd) NOT 'giardia'/exp AND ([embase]/lim OR [embase classic]/lim) AND [humans]/lim AND [english]/lim AND [abstracts]/lim) NOT 'helminth'/exp) NOT 'human immunodeficiency virus'/exp) AND 'protozoa'/exp | 2 | 3 Feb 2016 |
| #49 | 'protozoa'/exp | 38783 | 3 Feb 2016 |
| #48 | ((((((((((((((((('africa'/exp OR comoros OR djibouti OR madagascar OR malawi OR seychelles OR cameroon OR 'central african republic' OR chad OR congo OR 'equatorial guinea' OR 'atlantic islands' OR gabon OR morocco OR 'south sudan' OR sudan OR botswana OR lesotho OR swaziland OR benin OR 'burkina faso' OR 'cape verde' OR ghana OR guinea OR 'guinea-bissau' OR mauritania OR niger OR senegal OR 'sierra leone' OR togo OR 'burundi*' OR 'eritrea*' OR 'ethiopia*' OR 'kenya*' OR 'mozambique*' OR 'rwanda*' OR 'somalia*' OR 'tanzania*' OR 'uganda*' OR 'zambia*' OR 'zimbabwe*' OR 'angola*' OR 'algeria*' OR 'egypt*' OR 'tunisia*' OR 'namibia*' OR 'south africa*' OR 'gambia*' OR 'liberia*' OR 'mali*' OR 'nigeria*') AND ('anti-bacterial resistance' OR 'antibiotic resistan*' OR 'antimicrobial resistan*' OR 'drug resistan*' OR 'multi-drug resistan*' OR 'multidrug resistan*' OR 'multiple drug resistan*' OR 'antibiotic susceptib*' OR 'antimicrobial* susceptib*' OR 'drug* susceptib*' OR 'multi-drug susceptib*' OR 'multidrug susceptib*' OR 'multiple-drug susceptib*' OR 'multiple drug* susceptib*')) NOT 'antimalarial'/exp) NOT 'antituberculosis agent'/exp) NOT 'virus'/exp) NOT 'fungus'/exp) NOT 'malaria'/exp) NOT 'tuberculosis'/exp) NOT 'hiv'/exp) NOT 'cancer'/exp) NOT 'vaccine'/exp) NOT 'drug trial'/exp) NOT 'parasite'/exp) NOT cancer) NOT molecular AND [1-1-2013]/sd NOT [3-2-2016]/sd) NOT 'giardia'/exp AND ([embase]/lim OR [embase classic]/lim) AND [humans]/lim AND [english]/lim AND [abstracts]/lim) NOT 'helminth'/exp) NOT 'human immunodeficiency virus'/exp | 1095 | 3 Feb 2016 |
| #46 | 'human immunodeficiency virus'/exp | 155584 | 3 Feb 2016 |
| #45 | (((((((((((((((('africa'/exp OR comoros OR djibouti OR madagascar OR malawi OR seychelles OR cameroon OR 'central african republic' OR chad OR congo OR 'equatorial guinea' OR 'atlantic islands' OR gabon OR morocco OR 'south sudan' OR sudan OR botswana OR lesotho OR swaziland OR benin OR 'burkina faso' OR 'cape verde' OR ghana OR guinea OR 'guinea-bissau' OR mauritania OR niger OR senegal OR 'sierra leone' OR togo OR 'burundi*' OR 'eritrea*' OR 'ethiopia*' OR 'kenya*' OR 'mozambique*' OR 'rwanda*' OR 'somalia*' OR 'tanzania*' OR 'uganda*' OR 'zambia*' OR 'zimbabwe*' OR 'angola*' OR 'algeria*' OR 'egypt*' OR 'tunisia*' OR 'namibia*' OR 'south africa*' OR 'gambia*' OR 'liberia*' OR 'mali*' OR 'nigeria*') AND ('anti-bacterial resistance' OR 'antibiotic resistan*' OR 'antimicrobial resistan*' OR 'drug resistan*' OR 'multi-drug resistan*' OR 'multidrug resistan*' OR 'multiple drug resistan*' OR 'antibiotic susceptib*' OR 'antimicrobial* susceptib*' OR 'drug* susceptib*' OR 'multi-drug susceptib*' OR 'multidrug susceptib*' OR 'multiple-drug susceptib*' OR 'multiple drug* susceptib*')) NOT 'antimalarial'/exp) NOT 'antituberculosis agent'/exp) NOT 'virus'/exp) NOT 'fungus'/exp) NOT 'malaria'/exp) NOT 'tuberculosis'/exp) NOT 'hiv'/exp) NOT 'cancer'/exp) NOT 'vaccine'/exp) NOT 'drug trial'/exp) NOT 'parasite'/exp) NOT cancer) NOT molecular AND [1-1-2013]/sd NOT [3-2-2016]/sd) NOT 'giardia'/exp AND ([embase]/lim OR [embase classic]/lim) AND [humans]/lim AND [english]/lim AND [abstracts]/lim) NOT 'helminth'/exp | 1095 | 3 Feb 2016 |
| #43 | 'helminth'/exp | 152770 | 3 Feb 2016 |
| #42 | ((((((((((((((('africa'/exp OR comoros OR djibouti OR madagascar OR malawi OR seychelles OR cameroon OR 'central african republic' OR chad OR congo OR 'equatorial guinea' OR 'atlantic islands' OR gabon OR morocco OR 'south sudan' OR sudan OR botswana OR lesotho OR swaziland OR benin OR 'burkina faso' OR 'cape verde' OR ghana OR guinea OR 'guinea-bissau' OR mauritania OR niger OR senegal OR 'sierra leone' OR togo OR 'burundi*' OR 'eritrea*' OR 'ethiopia*' OR 'kenya*' OR 'mozambique*' OR 'rwanda*' OR 'somalia*' OR 'tanzania*' OR 'uganda*' OR 'zambia*' OR 'zimbabwe*' OR 'angola*' OR 'algeria*' OR 'egypt*' OR 'tunisia*' OR 'namibia*' OR 'south africa*' OR 'gambia*' OR 'liberia*' OR 'mali*' OR 'nigeria*') AND ('anti-bacterial resistance' OR 'antibiotic resistan*' OR 'antimicrobial resistan*' OR 'drug resistan*' OR 'multi-drug resistan*' OR 'multidrug resistan*' OR 'multiple drug resistan*' OR 'antibiotic susceptib*' OR 'antimicrobial* susceptib*' OR 'drug* susceptib*' OR 'multi-drug susceptib*' OR 'multidrug susceptib*' OR 'multiple-drug susceptib*' OR 'multiple drug* susceptib*')) NOT 'antimalarial'/exp) NOT 'antituberculosis agent'/exp) NOT 'virus'/exp) NOT 'fungus'/exp) NOT 'malaria'/exp) NOT 'tuberculosis'/exp) NOT 'hiv'/exp) NOT 'cancer'/exp) NOT 'vaccine'/exp) NOT 'drug trial'/exp) NOT 'parasite'/exp) NOT cancer) NOT molecular AND [1-1-2013]/sd NOT [3-2-2016]/sd) NOT 'giardia'/exp AND ([embase]/lim OR [embase classic]/lim) AND [humans]/lim AND [english]/lim AND [abstracts]/lim | 1107 | 3 Feb 2016 |
| #41 | ((((((((((((((('africa'/exp OR comoros OR djibouti OR madagascar OR malawi OR seychelles OR cameroon OR 'central african republic' OR chad OR congo OR 'equatorial guinea' OR 'atlantic islands' OR gabon OR morocco OR 'south sudan' OR sudan OR botswana OR lesotho OR swaziland OR benin OR 'burkina faso' OR 'cape verde' OR ghana OR guinea OR 'guinea-bissau' OR mauritania OR niger OR senegal OR 'sierra leone' OR togo OR 'burundi*' OR 'eritrea*' OR 'ethiopia*' OR 'kenya*' OR 'mozambique*' OR 'rwanda*' OR 'somalia*' OR 'tanzania*' OR 'uganda*' OR 'zambia*' OR 'zimbabwe*' OR 'angola*' OR 'algeria*' OR 'egypt*' OR 'tunisia*' OR 'namibia*' OR 'south africa*' OR 'gambia*' OR 'liberia*' OR 'mali*' OR 'nigeria*') AND ('anti-bacterial resistance' OR 'antibiotic resistan*' OR 'antimicrobial resistan*' OR 'drug resistan*' OR 'multi-drug resistan*' OR 'multidrug resistan*' OR 'multiple drug resistan*' OR 'antibiotic susceptib*' OR 'antimicrobial* susceptib*' OR 'drug* susceptib*' OR 'multi-drug susceptib*' OR 'multidrug susceptib*' OR 'multiple-drug susceptib*' OR 'multiple drug* susceptib*')) NOT 'antimalarial'/exp) NOT 'antituberculosis agent'/exp) NOT 'virus'/exp) NOT 'fungus'/exp) NOT 'malaria'/exp) NOT 'tuberculosis'/exp) NOT 'hiv'/exp) NOT 'cancer'/exp) NOT 'vaccine'/exp) NOT 'drug trial'/exp) NOT 'parasite'/exp) NOT cancer) NOT molecular AND [1-1-2013]/sd NOT [3-2-2016]/sd) NOT 'giardia'/exp AND ([embase]/lim OR [embase classic]/lim) AND [article in press]/lim AND [humans]/lim AND [english]/lim AND [abstracts]/lim | 23 | 3 Feb 2016 |
| #40 | ((((((((((((((('africa'/exp OR comoros OR djibouti OR madagascar OR malawi OR seychelles OR cameroon OR 'central african republic' OR chad OR congo OR 'equatorial guinea' OR 'atlantic islands' OR gabon OR morocco OR 'south sudan' OR sudan OR botswana OR lesotho OR swaziland OR benin OR 'burkina faso' OR 'cape verde' OR ghana OR guinea OR 'guinea-bissau' OR mauritania OR niger OR senegal OR 'sierra leone' OR togo OR 'burundi*' OR 'eritrea*' OR 'ethiopia*' OR 'kenya*' OR 'mozambique*' OR 'rwanda*' OR 'somalia*' OR 'tanzania*' OR 'uganda*' OR 'zambia*' OR 'zimbabwe*' OR 'angola*' OR 'algeria*' OR 'egypt*' OR 'tunisia*' OR 'namibia*' OR 'south africa*' OR 'gambia*' OR 'liberia*' OR 'mali*' OR 'nigeria*') AND ('anti-bacterial resistance' OR 'antibiotic resistan*' OR 'antimicrobial resistan*' OR 'drug resistan*' OR 'multi-drug resistan*' OR 'multidrug resistan*' OR 'multiple drug resistan*' OR 'antibiotic susceptib*' OR 'antimicrobial* susceptib*' OR 'drug* susceptib*' OR 'multi-drug susceptib*' OR 'multidrug susceptib*' OR 'multiple-drug susceptib*' OR 'multiple drug* susceptib*')) NOT 'antimalarial'/exp) NOT 'antituberculosis agent'/exp) NOT 'virus'/exp) NOT 'fungus'/exp) NOT 'malaria'/exp) NOT 'tuberculosis'/exp) NOT 'hiv'/exp) NOT 'cancer'/exp) NOT 'vaccine'/exp) NOT 'drug trial'/exp) NOT 'parasite'/exp) NOT cancer) NOT molecular AND [1-1-2013]/sd NOT [3-2-2016]/sd) NOT 'giardia'/exp AND ([embase]/lim OR [embase classic]/lim) | 1664 | 3 Feb 2016 |
| #39 | ((((((((((((((('africa'/exp OR comoros OR djibouti OR madagascar OR malawi OR seychelles OR cameroon OR 'central african republic' OR chad OR congo OR 'equatorial guinea' OR 'atlantic islands' OR gabon OR morocco OR 'south sudan' OR sudan OR botswana OR lesotho OR swaziland OR benin OR 'burkina faso' OR 'cape verde' OR ghana OR guinea OR 'guinea-bissau' OR mauritania OR niger OR senegal OR 'sierra leone' OR togo OR 'burundi*' OR 'eritrea*' OR 'ethiopia*' OR 'kenya*' OR 'mozambique*' OR 'rwanda*' OR 'somalia*' OR 'tanzania*' OR 'uganda*' OR 'zambia*' OR 'zimbabwe*' OR 'angola*' OR 'algeria*' OR 'egypt*' OR 'tunisia*' OR 'namibia*' OR 'south africa*' OR 'gambia*' OR 'liberia*' OR 'mali*' OR 'nigeria*') AND ('anti-bacterial resistance' OR 'antibiotic resistan*' OR 'antimicrobial resistan*' OR 'drug resistan*' OR 'multi-drug resistan*' OR 'multidrug resistan*' OR 'multiple drug resistan*' OR 'antibiotic susceptib*' OR 'antimicrobial* susceptib*' OR 'drug* susceptib*' OR 'multi-drug susceptib*' OR 'multidrug susceptib*' OR 'multiple-drug susceptib*' OR 'multiple drug* susceptib*')) NOT 'antimalarial'/exp) NOT 'antituberculosis agent'/exp) NOT 'virus'/exp) NOT 'fungus'/exp) NOT 'malaria'/exp) NOT 'tuberculosis'/exp) NOT 'hiv'/exp) NOT 'cancer'/exp) NOT 'vaccine'/exp) NOT 'drug trial'/exp) NOT 'parasite'/exp) NOT cancer) NOT molecular AND [1-1-2013]/sd NOT [3-2-2016]/sd) NOT 'giardia'/exp | 1865 | 3 Feb 2016 |
| #37 | 'giardia'/exp | 8566 | 3 Feb 2016 |
| #36 | (((((((((((((('africa'/exp OR comoros OR djibouti OR madagascar OR malawi OR seychelles OR cameroon OR 'central african republic' OR chad OR congo OR 'equatorial guinea' OR 'atlantic islands' OR gabon OR morocco OR 'south sudan' OR sudan OR botswana OR lesotho OR swaziland OR benin OR 'burkina faso' OR 'cape verde' OR ghana OR guinea OR 'guinea-bissau' OR mauritania OR niger OR senegal OR 'sierra leone' OR togo OR 'burundi*' OR 'eritrea*' OR 'ethiopia*' OR 'kenya*' OR 'mozambique*' OR 'rwanda*' OR 'somalia*' OR 'tanzania*' OR 'uganda*' OR 'zambia*' OR 'zimbabwe*' OR 'angola*' OR 'algeria*' OR 'egypt*' OR 'tunisia*' OR 'namibia*' OR 'south africa*' OR 'gambia*' OR 'liberia*' OR 'mali*' OR 'nigeria*') AND ('anti-bacterial resistance' OR 'antibiotic resistan*' OR 'antimicrobial resistan*' OR 'drug resistan*' OR 'multi-drug resistan*' OR 'multidrug resistan*' OR 'multiple drug resistan*' OR 'antibiotic susceptib*' OR 'antimicrobial* susceptib*' OR 'drug* susceptib*' OR 'multi-drug susceptib*' OR 'multidrug susceptib*' OR 'multiple-drug susceptib*' OR 'multiple drug* susceptib*')) NOT 'antimalarial'/exp) NOT 'antituberculosis agent'/exp) NOT 'virus'/exp) NOT 'fungus'/exp) NOT 'malaria'/exp) NOT 'tuberculosis'/exp) NOT 'hiv'/exp) NOT 'cancer'/exp) NOT 'vaccine'/exp) NOT 'drug trial'/exp) NOT 'parasite'/exp) NOT cancer) NOT molecular AND [1-1-2013]/sd NOT [3-2-2016]/sd | 1867 | 3 Feb 2016 |
| #35 | (((((((((((((('africa'/exp OR comoros OR djibouti OR madagascar OR malawi OR seychelles OR cameroon OR 'central african republic' OR chad OR congo OR 'equatorial guinea' OR 'atlantic islands' OR gabon OR morocco OR 'south sudan' OR sudan OR botswana OR lesotho OR swaziland OR benin OR 'burkina faso' OR 'cape verde' OR ghana OR guinea OR 'guinea-bissau' OR mauritania OR niger OR senegal OR 'sierra leone' OR togo OR 'burundi*' OR 'eritrea*' OR 'ethiopia*' OR 'kenya*' OR 'mozambique*' OR 'rwanda*' OR 'somalia*' OR 'tanzania*' OR 'uganda*' OR 'zambia*' OR 'zimbabwe*' OR 'angola*' OR 'algeria*' OR 'egypt*' OR 'tunisia*' OR 'namibia*' OR 'south africa*' OR 'gambia*' OR 'liberia*' OR 'mali*' OR 'nigeria*') AND ('anti-bacterial resistance' OR 'antibiotic resistan*' OR 'antimicrobial resistan*' OR 'drug resistan*' OR 'multi-drug resistan*' OR 'multidrug resistan*' OR 'multiple drug resistan*' OR 'antibiotic susceptib*' OR 'antimicrobial* susceptib*' OR 'drug* susceptib*' OR 'multi-drug susceptib*' OR 'multidrug susceptib*' OR 'multiple-drug susceptib*' OR 'multiple drug* susceptib*')) NOT 'antimalarial'/exp) NOT 'antituberculosis agent'/exp) NOT 'virus'/exp) NOT 'fungus'/exp) NOT 'malaria'/exp) NOT 'tuberculosis'/exp) NOT 'hiv'/exp) NOT 'cancer'/exp) NOT 'vaccine'/exp) NOT 'drug trial'/exp) NOT 'parasite'/exp) NOT cancer) NOT molecular | 7032 | 3 Feb 2016 |
| #34 | (((((((((((((('africa'/exp OR comoros OR djibouti OR madagascar OR malawi OR seychelles OR cameroon OR 'central african republic' OR chad OR congo OR 'equatorial guinea' OR 'atlantic islands' OR gabon OR morocco OR 'south sudan' OR sudan OR botswana OR lesotho OR swaziland OR benin OR 'burkina faso' OR 'cape verde' OR ghana OR guinea OR 'guinea-bissau' OR mauritania OR niger OR senegal OR 'sierra leone' OR togo OR 'burundi*' OR 'eritrea*' OR 'ethiopia*' OR 'kenya*' OR 'mozambique*' OR 'rwanda*' OR 'somalia*' OR 'tanzania*' OR 'uganda*' OR 'zambia*' OR 'zimbabwe*' OR 'angola*' OR 'algeria*' OR 'egypt*' OR 'tunisia*' OR 'namibia*' OR 'south africa*' OR 'gambia*' OR 'liberia*' OR 'mali*' OR 'nigeria*') AND ('anti-bacterial resistance' OR 'antibiotic resistan*' OR 'antimicrobial resistan*' OR 'drug resistan*' OR 'multi-drug resistan*' OR 'multidrug resistan*' OR 'multiple drug resistan*' OR 'antibiotic susceptib*' OR 'antimicrobial* susceptib*' OR 'drug* susceptib*' OR 'multi-drug susceptib*' OR 'multidrug susceptib*' OR 'multiple-drug susceptib*' OR 'multiple drug* susceptib*')) NOT 'antimalarial'/exp) NOT 'antituberculosis agent'/exp) NOT 'virus'/exp) NOT 'fungus'/exp) NOT 'malaria'/exp) NOT 'tuberculosis'/exp) NOT 'hiv'/exp) NOT 'cancer'/exp) NOT 'vaccine'/exp) NOT 'drug trial'/exp) NOT 'parasite'/exp) NOT cancer) AND molecular | 1202 | 3 Feb 2016 |
| #33 | molecular | 2836901 | 3 Feb 2016 |
| #32 | ((((((((((((('africa'/exp OR comoros OR djibouti OR madagascar OR malawi OR seychelles OR cameroon OR 'central african republic' OR chad OR congo OR 'equatorial guinea' OR 'atlantic islands' OR gabon OR morocco OR 'south sudan' OR sudan OR botswana OR lesotho OR swaziland OR benin OR 'burkina faso' OR 'cape verde' OR ghana OR guinea OR 'guinea-bissau' OR mauritania OR niger OR senegal OR 'sierra leone' OR togo OR 'burundi*' OR 'eritrea*' OR 'ethiopia*' OR 'kenya*' OR 'mozambique*' OR 'rwanda*' OR 'somalia*' OR 'tanzania*' OR 'uganda*' OR 'zambia*' OR 'zimbabwe*' OR 'angola*' OR 'algeria*' OR 'egypt*' OR 'tunisia*' OR 'namibia*' OR 'south africa*' OR 'gambia*' OR 'liberia*' OR 'mali*' OR 'nigeria*') AND ('anti-bacterial resistance' OR 'antibiotic resistan*' OR 'antimicrobial resistan*' OR 'drug resistan*' OR 'multi-drug resistan*' OR 'multidrug resistan*' OR 'multiple drug resistan*' OR 'antibiotic susceptib*' OR 'antimicrobial* susceptib*' OR 'drug* susceptib*' OR 'multi-drug susceptib*' OR 'multidrug susceptib*' OR 'multiple-drug susceptib*' OR 'multiple drug* susceptib*')) NOT 'antimalarial'/exp) NOT 'antituberculosis agent'/exp) NOT 'virus'/exp) NOT 'fungus'/exp) NOT 'malaria'/exp) NOT 'tuberculosis'/exp) NOT 'hiv'/exp) NOT 'cancer'/exp) NOT 'vaccine'/exp) NOT 'drug trial'/exp) NOT 'parasite'/exp) NOT cancer | 8234 | 3 Feb 2016 |
| #31 | ((((((((((((('africa'/exp OR comoros OR djibouti OR madagascar OR malawi OR seychelles OR cameroon OR 'central african republic' OR chad OR congo OR 'equatorial guinea' OR 'atlantic islands' OR gabon OR morocco OR 'south sudan' OR sudan OR botswana OR lesotho OR swaziland OR benin OR 'burkina faso' OR 'cape verde' OR ghana OR guinea OR 'guinea-bissau' OR mauritania OR niger OR senegal OR 'sierra leone' OR togo OR 'burundi*' OR 'eritrea*' OR 'ethiopia*' OR 'kenya*' OR 'mozambique*' OR 'rwanda*' OR 'somalia*' OR 'tanzania*' OR 'uganda*' OR 'zambia*' OR 'zimbabwe*' OR 'angola*' OR 'algeria*' OR 'egypt*' OR 'tunisia*' OR 'namibia*' OR 'south africa*' OR 'gambia*' OR 'liberia*' OR 'mali*' OR 'nigeria*') AND ('anti-bacterial resistance' OR 'antibiotic resistan*' OR 'antimicrobial resistan*' OR 'drug resistan*' OR 'multi-drug resistan*' OR 'multidrug resistan*' OR 'multiple drug resistan*' OR 'antibiotic susceptib*' OR 'antimicrobial* susceptib*' OR 'drug* susceptib*' OR 'multi-drug susceptib*' OR 'multidrug susceptib*' OR 'multiple-drug susceptib*' OR 'multiple drug* susceptib*')) NOT 'antimalarial'/exp) NOT 'antituberculosis agent'/exp) NOT 'virus'/exp) NOT 'fungus'/exp) NOT 'malaria'/exp) NOT 'tuberculosis'/exp) NOT 'hiv'/exp) NOT 'cancer'/exp) NOT 'vaccine'/exp) NOT 'drug trial'/exp) NOT 'parasite'/exp) AND cancer | 872 | 3 Feb 2016 |
| #30 | cancer | 3015954 | 3 Feb 2016 |
| #29 | (((((((((((('africa'/exp OR comoros OR djibouti OR madagascar OR malawi OR seychelles OR cameroon OR 'central african republic' OR chad OR congo OR 'equatorial guinea' OR 'atlantic islands' OR gabon OR morocco OR 'south sudan' OR sudan OR botswana OR lesotho OR swaziland OR benin OR 'burkina faso' OR 'cape verde' OR ghana OR guinea OR 'guinea-bissau' OR mauritania OR niger OR senegal OR 'sierra leone' OR togo OR 'burundi*' OR 'eritrea*' OR 'ethiopia*' OR 'kenya*' OR 'mozambique*' OR 'rwanda*' OR 'somalia*' OR 'tanzania*' OR 'uganda*' OR 'zambia*' OR 'zimbabwe*' OR 'angola*' OR 'algeria*' OR 'egypt*' OR 'tunisia*' OR 'namibia*' OR 'south africa*' OR 'gambia*' OR 'liberia*' OR 'mali*' OR 'nigeria*') AND ('anti-bacterial resistance' OR 'antibiotic resistan*' OR 'antimicrobial resistan*' OR 'drug resistan*' OR 'multi-drug resistan*' OR 'multidrug resistan*' OR 'multiple drug resistan*' OR 'antibiotic susceptib*' OR 'antimicrobial* susceptib*' OR 'drug* susceptib*' OR 'multi-drug susceptib*' OR 'multidrug susceptib*' OR 'multiple-drug susceptib*' OR 'multiple drug* susceptib*')) NOT 'antimalarial'/exp) NOT 'antituberculosis agent'/exp) NOT 'virus'/exp) NOT 'fungus'/exp) NOT 'malaria'/exp) NOT 'tuberculosis'/exp) NOT 'hiv'/exp) NOT 'cancer'/exp) NOT 'vaccine'/exp) NOT 'drug trial'/exp) NOT 'parasite'/exp | 9106 | 3 Feb 2016 |
| #28 | 'parasite'/exp | 29544 | 3 Feb 2016 |
| #27 | ((((((((((('africa'/exp OR comoros OR djibouti OR madagascar OR malawi OR seychelles OR cameroon OR 'central african republic' OR chad OR congo OR 'equatorial guinea' OR 'atlantic islands' OR gabon OR morocco OR 'south sudan' OR sudan OR botswana OR lesotho OR swaziland OR benin OR 'burkina faso' OR 'cape verde' OR ghana OR guinea OR 'guinea-bissau' OR mauritania OR niger OR senegal OR 'sierra leone' OR togo OR 'burundi*' OR 'eritrea*' OR 'ethiopia*' OR 'kenya*' OR 'mozambique*' OR 'rwanda*' OR 'somalia*' OR 'tanzania*' OR 'uganda*' OR 'zambia*' OR 'zimbabwe*' OR 'angola*' OR 'algeria*' OR 'egypt*' OR 'tunisia*' OR 'namibia*' OR 'south africa*' OR 'gambia*' OR 'liberia*' OR 'mali*' OR 'nigeria*') AND ('anti-bacterial resistance' OR 'antibiotic resistan*' OR 'antimicrobial resistan*' OR 'drug resistan*' OR 'multi-drug resistan*' OR 'multidrug resistan*' OR 'multiple drug resistan*' OR 'antibiotic susceptib*' OR 'antimicrobial* susceptib*' OR 'drug* susceptib*' OR 'multi-drug susceptib*' OR 'multidrug susceptib*' OR 'multiple-drug susceptib*' OR 'multiple drug* susceptib*')) NOT 'antimalarial'/exp) NOT 'antituberculosis agent'/exp) NOT 'virus'/exp) NOT 'fungus'/exp) NOT 'malaria'/exp) NOT 'tuberculosis'/exp) NOT 'hiv'/exp) NOT 'cancer'/exp) NOT 'vaccine'/exp) NOT 'drug trial'/exp | 9135 | 3 Feb 2016 |
| #26 | (((((((((('africa'/exp OR comoros OR djibouti OR madagascar OR malawi OR seychelles OR cameroon OR 'central african republic' OR chad OR congo OR 'equatorial guinea' OR 'atlantic islands' OR gabon OR morocco OR 'south sudan' OR sudan OR botswana OR lesotho OR swaziland OR benin OR 'burkina faso' OR 'cape verde' OR ghana OR guinea OR 'guinea-bissau' OR mauritania OR niger OR senegal OR 'sierra leone' OR togo OR 'burundi*' OR 'eritrea*' OR 'ethiopia*' OR 'kenya*' OR 'mozambique*' OR 'rwanda*' OR 'somalia*' OR 'tanzania*' OR 'uganda*' OR 'zambia*' OR 'zimbabwe*' OR 'angola*' OR 'algeria*' OR 'egypt*' OR 'tunisia*' OR 'namibia*' OR 'south africa*' OR 'gambia*' OR 'liberia*' OR 'mali*' OR 'nigeria*') AND ('anti-bacterial resistance' OR 'antibiotic resistan*' OR 'antimicrobial resistan*' OR 'drug resistan*' OR 'multi-drug resistan*' OR 'multidrug resistan*' OR 'multiple drug resistan*' OR 'antibiotic susceptib*' OR 'antimicrobial* susceptib*' OR 'drug* susceptib*' OR 'multi-drug susceptib*' OR 'multidrug susceptib*' OR 'multiple-drug susceptib*' OR 'multiple drug* susceptib*')) NOT 'antimalarial'/exp) NOT 'antituberculosis agent'/exp) NOT 'virus'/exp) NOT 'fungus'/exp) NOT 'malaria'/exp) NOT 'tuberculosis'/exp) NOT 'hiv'/exp) NOT 'cancer'/exp) NOT 'vaccine'/exp | 9289 | 3 Feb 2016 |
| #25 | ((((((((('africa'/exp OR comoros OR djibouti OR madagascar OR malawi OR seychelles OR cameroon OR 'central african republic' OR chad OR congo OR 'equatorial guinea' OR 'atlantic islands' OR gabon OR morocco OR 'south sudan' OR sudan OR botswana OR lesotho OR swaziland OR benin OR 'burkina faso' OR 'cape verde' OR ghana OR guinea OR 'guinea-bissau' OR mauritania OR niger OR senegal OR 'sierra leone' OR togo OR 'burundi*' OR 'eritrea*' OR 'ethiopia*' OR 'kenya*' OR 'mozambique*' OR 'rwanda*' OR 'somalia*' OR 'tanzania*' OR 'uganda*' OR 'zambia*' OR 'zimbabwe*' OR 'angola*' OR 'algeria*' OR 'egypt*' OR 'tunisia*' OR 'namibia*' OR 'south africa*' OR 'gambia*' OR 'liberia*' OR 'mali*' OR 'nigeria*') AND ('anti-bacterial resistance' OR 'antibiotic resistan*' OR 'antimicrobial resistan*' OR 'drug resistan*' OR 'multi-drug resistan*' OR 'multidrug resistan*' OR 'multiple drug resistan*' OR 'antibiotic susceptib*' OR 'antimicrobial* susceptib*' OR 'drug* susceptib*' OR 'multi-drug susceptib*' OR 'multidrug susceptib*' OR 'multiple-drug susceptib*' OR 'multiple drug* susceptib*')) NOT 'antimalarial'/exp) NOT 'antituberculosis agent'/exp) NOT 'virus'/exp) NOT 'fungus'/exp) NOT 'malaria'/exp) NOT 'tuberculosis'/exp) NOT 'hiv'/exp) NOT 'cancer'/exp | 9590 | 3 Feb 2016 |
| #24 | (((((((('africa'/exp OR comoros OR djibouti OR madagascar OR malawi OR seychelles OR cameroon OR 'central african republic' OR chad OR congo OR 'equatorial guinea' OR 'atlantic islands' OR gabon OR morocco OR 'south sudan' OR sudan OR botswana OR lesotho OR swaziland OR benin OR 'burkina faso' OR 'cape verde' OR ghana OR guinea OR 'guinea-bissau' OR mauritania OR niger OR senegal OR 'sierra leone' OR togo OR 'burundi*' OR 'eritrea*' OR 'ethiopia*' OR 'kenya*' OR 'mozambique*' OR 'rwanda*' OR 'somalia*' OR 'tanzania*' OR 'uganda*' OR 'zambia*' OR 'zimbabwe*' OR 'angola*' OR 'algeria*' OR 'egypt*' OR 'tunisia*' OR 'namibia*' OR 'south africa*' OR 'gambia*' OR 'liberia*' OR 'mali*' OR 'nigeria*') AND ('anti-bacterial resistance' OR 'antibiotic resistan*' OR 'antimicrobial resistan*' OR 'drug resistan*' OR 'multi-drug resistan*' OR 'multidrug resistan*' OR 'multiple drug resistan*' OR 'antibiotic susceptib*' OR 'antimicrobial* susceptib*' OR 'drug* susceptib*' OR 'multi-drug susceptib*' OR 'multidrug susceptib*' OR 'multiple-drug susceptib*' OR 'multiple drug* susceptib*')) NOT 'antimalarial'/exp) NOT 'antituberculosis agent'/exp) NOT 'virus'/exp) NOT 'fungus'/exp) NOT 'malaria'/exp) NOT 'tuberculosis'/exp) NOT 'hiv'/exp | 19846 | 3 Feb 2016 |
| #22 | ((((((('africa'/exp OR comoros OR djibouti OR madagascar OR malawi OR seychelles OR cameroon OR 'central african republic' OR chad OR congo OR 'equatorial guinea' OR 'atlantic islands' OR gabon OR morocco OR 'south sudan' OR sudan OR botswana OR lesotho OR swaziland OR benin OR 'burkina faso' OR 'cape verde' OR ghana OR guinea OR 'guinea-bissau' OR mauritania OR niger OR senegal OR 'sierra leone' OR togo OR 'burundi*' OR 'eritrea*' OR 'ethiopia*' OR 'kenya*' OR 'mozambique*' OR 'rwanda*' OR 'somalia*' OR 'tanzania*' OR 'uganda*' OR 'zambia*' OR 'zimbabwe*' OR 'angola*' OR 'algeria*' OR 'egypt*' OR 'tunisia*' OR 'namibia*' OR 'south africa*' OR 'gambia*' OR 'liberia*' OR 'mali*' OR 'nigeria*') AND ('anti-bacterial resistance' OR 'antibiotic resistan*' OR 'antimicrobial resistan*' OR 'drug resistan*' OR 'multi-drug resistan*' OR 'multidrug resistan*' OR 'multiple drug resistan*' OR 'antibiotic susceptib*' OR 'antimicrobial* susceptib*' OR 'drug* susceptib*' OR 'multi-drug susceptib*' OR 'multidrug susceptib*' OR 'multiple-drug susceptib*' OR 'multiple drug* susceptib*')) NOT 'antimalarial'/exp) NOT 'antituberculosis agent'/exp) NOT 'virus'/exp) NOT 'fungus'/exp) NOT 'malaria'/exp) NOT 'tuberculosis'/exp | 19846 | 3 Feb 2016 |
| #21 | ((((((('africa'/exp OR comoros OR djibouti OR madagascar OR malawi OR seychelles OR cameroon OR 'central african republic' OR chad OR congo OR 'equatorial guinea' OR 'atlantic islands' OR gabon OR morocco OR 'south sudan' OR sudan OR botswana OR lesotho OR swaziland OR benin OR 'burkina faso' OR 'cape verde' OR ghana OR guinea OR 'guinea-bissau' OR mauritania OR niger OR senegal OR 'sierra leone' OR togo OR 'burundi*' OR 'eritrea*' OR 'ethiopia*' OR 'kenya*' OR 'mozambique*' OR 'rwanda*' OR 'somalia*' OR 'tanzania*' OR 'uganda*' OR 'zambia*' OR 'zimbabwe*' OR 'angola*' OR 'algeria*' OR 'egypt*' OR 'tunisia*' OR 'namibia*' OR 'south africa*' OR 'gambia*' OR 'liberia*' OR 'mali*' OR 'nigeria*') AND ('anti-bacterial resistance' OR 'antibiotic resistan*' OR 'antimicrobial resistan*' OR 'drug resistan*' OR 'multi-drug resistan*' OR 'multidrug resistan*' OR 'multiple drug resistan*' OR 'antibiotic susceptib*' OR 'antimicrobial* susceptib*' OR 'drug* susceptib*' OR 'multi-drug susceptib*' OR 'multidrug susceptib*' OR 'multiple-drug susceptib*' OR 'multiple drug* susceptib*')) NOT 'antimalarial'/exp) NOT 'antituberculosis agent'/exp) NOT 'virus'/exp) NOT 'fungus'/exp) NOT 'malaria'/exp) NOT 'fungus'/exp | 20633 | 3 Feb 2016 |
| #20 | (((((('africa'/exp OR comoros OR djibouti OR madagascar OR malawi OR seychelles OR cameroon OR 'central african republic' OR chad OR congo OR 'equatorial guinea' OR 'atlantic islands' OR gabon OR morocco OR 'south sudan' OR sudan OR botswana OR lesotho OR swaziland OR benin OR 'burkina faso' OR 'cape verde' OR ghana OR guinea OR 'guinea-bissau' OR mauritania OR niger OR senegal OR 'sierra leone' OR togo OR 'burundi*' OR 'eritrea*' OR 'ethiopia*' OR 'kenya*' OR 'mozambique*' OR 'rwanda*' OR 'somalia*' OR 'tanzania*' OR 'uganda*' OR 'zambia*' OR 'zimbabwe*' OR 'angola*' OR 'algeria*' OR 'egypt*' OR 'tunisia*' OR 'namibia*' OR 'south africa*' OR 'gambia*' OR 'liberia*' OR 'mali*' OR 'nigeria*') AND ('anti-bacterial resistance' OR 'antibiotic resistan*' OR 'antimicrobial resistan*' OR 'drug resistan*' OR 'multi-drug resistan*' OR 'multidrug resistan*' OR 'multiple drug resistan*' OR 'antibiotic susceptib*' OR 'antimicrobial* susceptib*' OR 'drug* susceptib*' OR 'multi-drug susceptib*' OR 'multidrug susceptib*' OR 'multiple-drug susceptib*' OR 'multiple drug* susceptib*')) NOT 'antimalarial'/exp) NOT 'antituberculosis agent'/exp) NOT 'virus'/exp) NOT 'fungus'/exp) NOT 'malaria'/exp | 20633 | 3 Feb 2016 |
| #19 | ((((('africa'/exp OR comoros OR djibouti OR madagascar OR malawi OR seychelles OR cameroon OR 'central african republic' OR chad OR congo OR 'equatorial guinea' OR 'atlantic islands' OR gabon OR morocco OR 'south sudan' OR sudan OR botswana OR lesotho OR swaziland OR benin OR 'burkina faso' OR 'cape verde' OR ghana OR guinea OR 'guinea-bissau' OR mauritania OR niger OR senegal OR 'sierra leone' OR togo OR 'burundi*' OR 'eritrea*' OR 'ethiopia*' OR 'kenya*' OR 'mozambique*' OR 'rwanda*' OR 'somalia*' OR 'tanzania*' OR 'uganda*' OR 'zambia*' OR 'zimbabwe*' OR 'angola*' OR 'algeria*' OR 'egypt*' OR 'tunisia*' OR 'namibia*' OR 'south africa*' OR 'gambia*' OR 'liberia*' OR 'mali*' OR 'nigeria*') AND ('anti-bacterial resistance' OR 'antibiotic resistan*' OR 'antimicrobial resistan*' OR 'drug resistan*' OR 'multi-drug resistan*' OR 'multidrug resistan*' OR 'multiple drug resistan*' OR 'antibiotic susceptib*' OR 'antimicrobial* susceptib*' OR 'drug* susceptib*' OR 'multi-drug susceptib*' OR 'multidrug susceptib*' OR 'multiple-drug susceptib*' OR 'multiple drug* susceptib*')) NOT 'antimalarial'/exp) NOT 'antituberculosis agent'/exp) NOT 'virus'/exp) NOT 'fungus'/exp | 20954 | 3 Feb 2016 |
| #18 | (((('africa'/exp OR comoros OR djibouti OR madagascar OR malawi OR seychelles OR cameroon OR 'central african republic' OR chad OR congo OR 'equatorial guinea' OR 'atlantic islands' OR gabon OR morocco OR 'south sudan' OR sudan OR botswana OR lesotho OR swaziland OR benin OR 'burkina faso' OR 'cape verde' OR ghana OR guinea OR 'guinea-bissau' OR mauritania OR niger OR senegal OR 'sierra leone' OR togo OR 'burundi*' OR 'eritrea*' OR 'ethiopia*' OR 'kenya*' OR 'mozambique*' OR 'rwanda*' OR 'somalia*' OR 'tanzania*' OR 'uganda*' OR 'zambia*' OR 'zimbabwe*' OR 'angola*' OR 'algeria*' OR 'egypt*' OR 'tunisia*' OR 'namibia*' OR 'south africa*' OR 'gambia*' OR 'liberia*' OR 'mali*' OR 'nigeria*') AND ('anti-bacterial resistance' OR 'antibiotic resistan*' OR 'antimicrobial resistan*' OR 'drug resistan*' OR 'multi-drug resistan*' OR 'multidrug resistan*' OR 'multiple drug resistan*' OR 'antibiotic susceptib*' OR 'antimicrobial* susceptib*' OR 'drug* susceptib*' OR 'multi-drug susceptib*' OR 'multidrug susceptib*' OR 'multiple-drug susceptib*' OR 'multiple drug* susceptib*')) NOT 'antimalarial'/exp) NOT 'antituberculosis agent'/exp) NOT 'virus'/exp | 21783 | 3 Feb 2016 |
| #17 | ((('africa'/exp OR comoros OR djibouti OR madagascar OR malawi OR seychelles OR cameroon OR 'central african republic' OR chad OR congo OR 'equatorial guinea' OR 'atlantic islands' OR gabon OR morocco OR 'south sudan' OR sudan OR botswana OR lesotho OR swaziland OR benin OR 'burkina faso' OR 'cape verde' OR ghana OR guinea OR 'guinea-bissau' OR mauritania OR niger OR senegal OR 'sierra leone' OR togo OR 'burundi*' OR 'eritrea*' OR 'ethiopia*' OR 'kenya*' OR 'mozambique*' OR 'rwanda*' OR 'somalia*' OR 'tanzania*' OR 'uganda*' OR 'zambia*' OR 'zimbabwe*' OR 'angola*' OR 'algeria*' OR 'egypt*' OR 'tunisia*' OR 'namibia*' OR 'south africa*' OR 'gambia*' OR 'liberia*' OR 'mali*' OR 'nigeria*') AND ('anti-bacterial resistance' OR 'antibiotic resistan*' OR 'antimicrobial resistan*' OR 'drug resistan*' OR 'multi-drug resistan*' OR 'multidrug resistan*' OR 'multiple drug resistan*' OR 'antibiotic susceptib*' OR 'antimicrobial* susceptib*' OR 'drug* susceptib*' OR 'multi-drug susceptib*' OR 'multidrug susceptib*' OR 'multiple-drug susceptib*' OR 'multiple drug* susceptib*')) NOT 'antimalarial'/exp) NOT 'antituberculosis agent'/exp | 23792 | 3 Feb 2016 |
| #16 | (('africa'/exp OR comoros OR djibouti OR madagascar OR malawi OR seychelles OR cameroon OR 'central african republic' OR chad OR congo OR 'equatorial guinea' OR 'atlantic islands' OR gabon OR morocco OR 'south sudan' OR sudan OR botswana OR lesotho OR swaziland OR benin OR 'burkina faso' OR 'cape verde' OR ghana OR guinea OR 'guinea-bissau' OR mauritania OR niger OR senegal OR 'sierra leone' OR togo OR 'burundi*' OR 'eritrea*' OR 'ethiopia*' OR 'kenya*' OR 'mozambique*' OR 'rwanda*' OR 'somalia*' OR 'tanzania*' OR 'uganda*' OR 'zambia*' OR 'zimbabwe*' OR 'angola*' OR 'algeria*' OR 'egypt*' OR 'tunisia*' OR 'namibia*' OR 'south africa*' OR 'gambia*' OR 'liberia*' OR 'mali*' OR 'nigeria*') AND ('anti-bacterial resistance' OR 'antibiotic resistan*' OR 'antimicrobial resistan*' OR 'drug resistan*' OR 'multi-drug resistan*' OR 'multidrug resistan*' OR 'multiple drug resistan*' OR 'antibiotic susceptib*' OR 'antimicrobial* susceptib*' OR 'drug* susceptib*' OR 'multi-drug susceptib*' OR 'multidrug susceptib*' OR 'multiple-drug susceptib*' OR 'multiple drug* susceptib*')) NOT 'antimalarial'/exp | 26899 | 3 Feb 2016 |
| #15 | (('africa'/exp OR comoros OR djibouti OR madagascar OR malawi OR seychelles OR cameroon OR 'central african republic' OR chad OR congo OR 'equatorial guinea' OR 'atlantic islands' OR gabon OR morocco OR 'south sudan' OR sudan OR botswana OR lesotho OR swaziland OR benin OR 'burkina faso' OR 'cape verde' OR ghana OR guinea OR 'guinea-bissau' OR mauritania OR niger OR senegal OR 'sierra leone' OR togo OR 'burundi*' OR 'eritrea*' OR 'ethiopia*' OR 'kenya*' OR 'mozambique*' OR 'rwanda*' OR 'somalia*' OR 'tanzania*' OR 'uganda*' OR 'zambia*' OR 'zimbabwe*' OR 'angola*' OR 'algeria*' OR 'egypt*' OR 'tunisia*' OR 'namibia*' OR 'south africa*' OR 'gambia*' OR 'liberia*' OR 'mali*' OR 'nigeria*') AND ('anti-bacterial resistance' OR 'antibiotic resistan*' OR 'antimicrobial resistan*' OR 'drug resistan*' OR 'multi-drug resistan*' OR 'multidrug resistan*' OR 'multiple drug resistan*' OR 'antibiotic susceptib*' OR 'antimicrobial* susceptib*' OR 'drug* susceptib*' OR 'multi-drug susceptib*' OR 'multidrug susceptib*' OR 'multiple-drug susceptib*' OR 'multiple drug* susceptib*')) NOT ('antimalarial'/exp OR 'antituberculosis agent'/exp OR 'virus'/exp OR 'fungus'/exp OR 'malaria'/exp OR 'tuberculosis'/exp OR 'hiv'/exp OR 'cancer'/exp OR 'vaccine'/exp OR 'drug trial'/exp) | 9135 | 3 Feb 2016 |
| #14 | 'antimalarial'/exp OR 'antituberculosis agent'/exp OR 'virus'/exp OR 'fungus'/exp OR 'malaria'/exp OR 'tuberculosis'/exp OR 'hiv'/exp OR 'cancer'/exp OR 'vaccine'/exp OR 'drug trial'/exp | 5792200 | 3 Feb 2016 |
| #13 | 'drug trial'/exp | 148437 | 3 Feb 2016 |
| #12 | 'vaccine'/exp | 289834 | 3 Feb 2016 |
| #11 | 'cancer'/exp | 3902388 | 3 Feb 2016 |
| #10 | 'hiv'/exp | 155584 | 3 Feb 2016 |
| #9 | 'tuberculosis'/exp | 246048 | 3 Feb 2016 |
| #8 | 'malaria'/exp | 82934 | 3 Feb 2016 |
| #7 | 'fungus'/exp | 449689 | 3 Feb 2016 |
| #6 | 'virus'/exp | 923859 | 3 Feb 2016 |
| #5 | 'antituberculosis agent'/exp | 151160 | 3 Feb 2016 |
| #4 | 'antimalarial'/exp | 101984 | 3 Feb 2016 |
| #3 | ('africa'/exp OR comoros OR djibouti OR madagascar OR malawi OR seychelles OR cameroon OR 'central african republic' OR chad OR congo OR 'equatorial guinea' OR 'atlantic islands' OR gabon OR morocco OR 'south sudan' OR sudan OR botswana OR lesotho OR swaziland OR benin OR 'burkina faso' OR 'cape verde' OR ghana OR guinea OR 'guinea-bissau' OR mauritania OR niger OR senegal OR 'sierra leone' OR togo OR 'burundi*' OR 'eritrea*' OR 'ethiopia*' OR 'kenya*' OR 'mozambique*' OR 'rwanda*' OR 'somalia*' OR 'tanzania*' OR 'uganda*' OR 'zambia*' OR 'zimbabwe*' OR 'angola*' OR 'algeria*' OR 'egypt*' OR 'tunisia*' OR 'namibia*' OR 'south africa*' OR 'gambia*' OR 'liberia*' OR 'mali*' OR 'nigeria*') AND ('anti-bacterial resistance' OR 'antibiotic resistan*' OR 'antimicrobial resistan*' OR 'drug resistan*' OR 'multi-drug resistan*' OR 'multidrug resistan*' OR 'multiple drug resistan*' OR 'antibiotic susceptib*' OR 'antimicrobial* susceptib*' OR 'drug* susceptib*' OR 'multi-drug susceptib*' OR 'multidrug susceptib*' OR 'multiple-drug susceptib*' OR 'multiple drug* susceptib*') | 30375 | 3 Feb 2016 |
| #2 | 'anti-bacterial resistance' OR 'antibiotic resistan*' OR 'antimicrobial resistan*' OR 'drug resistan*' OR 'multi-drug resistan*' OR 'multidrug resistan*' OR 'multiple drug resistan*' OR 'antibiotic susceptib*' OR 'antimicrobial* susceptib*' OR 'drug* susceptib*' OR 'multi-drug susceptib*' OR 'multidrug susceptib*' OR 'multiple-drug susceptib*' OR 'multiple drug* susceptib*' | 350621 | 3 Feb 2016 |
| #1 | 'africa'/exp OR comoros OR djibouti OR madagascar OR malawi OR seychelles OR cameroon OR 'central african republic' OR chad OR congo OR 'equatorial guinea' OR 'atlantic islands' OR gabon OR morocco OR 'south sudan' OR sudan OR botswana OR lesotho OR swaziland OR benin OR 'burkina faso' OR 'cape verde' OR ghana OR guinea OR 'guinea-bissau' OR mauritania OR niger OR senegal OR 'sierra leone' OR togo OR 'burundi*' OR 'eritrea*' OR 'ethiopia*' OR 'kenya*' OR 'mozambique*' OR 'rwanda*' OR 'somalia*' OR 'tanzania*' OR 'uganda*' OR 'zambia*' OR 'zimbabwe*' OR 'angola*' OR 'algeria*' OR 'egypt*' OR 'tunisia*' OR 'namibia*' OR 'south africa*' OR 'gambia*' OR 'liberia*' OR 'mali*' OR 'nigeria*' | 1548119 | 3 Feb 2016 |

Search Strategy for Cochrane database (Februray 03, 2016):

“Bacteria*” OR “Antibiotic resistan*” OR “Antimicrobial* resistan*” OR “Drug* resistan*” OR “Multi-drug resistan*” OR “Multidrug resistan*” OR “Multiple-drug resistan*” OR “Multiple drug* resistan*” “Antibiotic* susceptib*” OR “Antimicrobial* susceptib*” OR “Drug* susceptib*” OR “Multi-drug susceptib*” OR “Multidrug susceptib*” OR “Multiple-drug susceptib*” OR “Multiple drug* susceptib*” AND “Africa*”

### #1 "Antibiotic resistan*" 1067

#2 "Antimicrobial* resistan*" 290

#3 "Drug* resistan*" 5924

#4 "Multidrug resistan*" 806

#5 "Multiple drug* resistan*" 84

#6 "Antibiotic* susceptib*" 154

#7 "Antimicrobial* susceptib*" 130

#8 "Drug* susceptib*" 149

#9 "Multi-drug susceptib*" 0

#10 "Multidrug susceptib*" 0

#11 "Multiple-drug susceptib*" 0

#12 "Multiple drug* susceptib*" 0

#13 "africa*" or Comoros or Djibouti or Madagascar or Malawi or Seychelles or Cameroon or "Central African Republic" or Chad or Congo or "Equatorial Guinea" or "Atlantic Islands" or Gabon or Morocco or "South Sudan" or Sudan or Botswana or Lesotho or Swaziland or Benin or "Burkina Faso" or "Cape Verde" or Ghana or Guinea or "Guinea-Bissau" or Mauritania or Niger or Senegal or "Sierra Leone" or Togo or "Burundi*" or "eritrea*" or "ethiopia*" or "kenya*" or "mozambique*" or "rwanda*" or "somalia*" or "tanzania*" or "uganda*" or "zambia*" or "zimbabwe*" or "angola*" or "algeria*" or "egypt*" or "tunisia*" or "namibia*" or "south africa*" or "gambia*" or "liberia*" or "mali*" or "nigeria*" 36165

#14 "vir*" 35706

#15 HIV 14604

#16 "tubercul*" 5105

#17 "malaria*" 3970

#18 neoplasm 16248

#19 "antimalaria*" 1990

#20 vaccine 11875

#21 "europ*" 75991

#22 "US*" 527703

#24 #1 or #2 or #3 or #4 or #5 or #6 or #7 or #8 or #9 or #10 or #11 or #12 7381

#25 #24 and #13 1027

#26 #25 not #14 720

#27 #26 not #15 655

#28 #27 not #16 603

#29 #28 not #17 259

#30 #29 not #18 226

#31 #30 not #19 224

#32 #31 not #20 218

#33 #32 not #21 169

#34 #33 not #22 32

Search strategy for Science Daily (February 03, 2016):

“Bacteria” “Antibiotic resistan*” “Antimicrobial* resistan*” “Drug* resistan*” “Multi-drug resistan*” “Multidrug resistan*” “Multiple-drug resistan*” “Multiple drug* resistan*” “Antibiotic* susceptib*” “Antimicrobial* susceptib*” “Drug* susceptib*” “Multi-drug susceptib*” “Multidrug susceptib*” “Multiple-drug susceptib*” “Multiple drug* susceptib*”

**Article Quality Assessment**

The quality of each article was assessed using a tool modified for the purposes of this study from criteria published by Omulo *et al.* and the Cochrane guidelines for assessing bias in observational studies. Since a limited number of articles was available, results of the quality assessment were not used for inclusion/exclusion. The quality criteria included 26 items to assess the design, details of sample collection, processing and storage, reporting on AMR methodologies and quality assurance strategies.

| Criteria | Number (Percent) |
| --- | --- |
| 1. Is the research design described? | 113 (78.5) |
| 1. Does the study indicate the months and years of the study and provide data on any potential confounding events (e.g., natural disasters) during the study period? | 134 (93.1) |
| 1. Does the study state the period of time during which samples were specifically collected? | 135 (93.8) |
| 1. Is the setting of the study and data acquisition clearly described; for example, hospital acquired versus community acquired? | 116 (80.6) |
| 1. Is the study population clearly described (example, percent children and percent adults, comorbidities? | 113 (78.5) |
| 1. Are the criteria for enrolment in the study clearly stated? | 98 (68.1) |
| 1. Is there a clear description of the types of specimen collected and how these were collected? | 131 (91) |
| 1. Did the study describe the overall and specific numbers of samples collected (by category sampled)? | 85 (59) |
| 1. Does the study describe the number of samples tested and indicate reasons for exclusion, if any? | 110 (76.4) |
| 1. Is there description of how specimens were handled, transported and stored after collection? | 86 (59.7) |
| 1. Is details of conditions of sample storage like temperature and place described? | 75 (52.1) |
| 1. Is the duration of sample storage described? | 70 (48.6) |
| 1. What is the number of samples that were tested (excluding discarded samples)? | 115 (79.9) |
| 1. Are the media for culture described? | 122 (84.7) |
| 1. Did the study describe the total number of isolates? | 128 (88.9) |
| 1. Does the study describe isolates by source? | 120 (83.3) |
| 1. Does the study describe isolates by category including sex and age? | 117 (81.3) |
| 1. Does the study describe the type of susceptibility testing used? | 126 (87.5) |
| 1. Did the study use any internal quality control measures (example, reference strains)? | 69 (47.9) |
| 1. Does the study describe how discordant results were resolved when more than one method was employed? | NA |
| 1. Did the study specify the testing standard used (e.g. CLSI, EUCAST) | 111 (77.1) |
| 1. Did the study indicate if quality testing procedures were used (e.g. confirmatory testing by independent lab) and concordance level? | 3 (2.1) |
| 1. Did the study calculate the frequency of resistance to an antibiotic as the total number of resistant isolates divided by the total number of isolates tested with a given antibiotic? | 136 (94.4) |
| 1. Did the study tabulate resistances for all antibiotics tested indicating the absolute numbers or resistant isolates, and the percent (%) of resistant isolates? | 137 (95.1) |
| 1. Did the study compare categories using appropriate statistics with an explicit description of how replicates were defined and how technical vs. independent replicates were processed? | 138 (95.8) |

NA: Not applicable (there were no studies which used two methods at the same time)
