# Supplementary material for: Antimicrobial resistance in Africa: a systematic review
Source: BMC Infect Dis. 2017 Sep 11;17:616. doi: 10.1186/s12879-017-2713-1 (PMC5594539; doi:10.1186/s12879-017-2713-1)
Supplement: Supplementary file 2 — Proportion of resistance of bacterial isolates to tested antibiotics by region and country (DOCX 263 kb) [file 12879_2017_2713_MOESM2_ESM.docx]

Additional file 2: Proportion of resistance of bacterial isolates to tested antibiotics by region and country

| **Country**  **(References)** | **Bacteria (Total number of isoaltes)** | **amk** | **amx** | **amp** | **aug** | **azi** | **cfz** | **cfp** | **ctx** | **cxt** | **caz** | **Cro** | **cfu** | **cpx** | **cpt** | **c** | **cip** | **cli** | **stx** |
| --- | --- | --- | --- | --- | --- | --- | --- | --- | --- | --- | --- | --- | --- | --- | --- | --- | --- | --- | --- |
| Djibouti | *Neisseria gonorrhoeae (24)* | ND | ND | ND | ND | 4.2 | ND | 12.5 | ND | ND | ND | 12.5 | ND | ND | ND | ND | 37.5 | ND | ND |
| Ethiopia | *Acinetobacter* spp*. (12)* | ND | ND | 57.1 | 20.0 | ND | ND | ND | 57.1 | ND | ND | 66.7 | ND | ND | ND | 57.1 | 8.3 | ND | 50.0 |
|  | *Citrobacter* spp*. (80)* | ND | 73.7 | 88.1 | 34.8 | 50.0 | ND | 0.0 | 29.2 | ND | 40.0 | 25.3 | ND | ND | ND | 70.8 | 20.0 | ND | 61.3 |
|  | *CoNS (353)* | 0.0 | ND | 77.8 | 38.5 | 61.8 | ND | ND | 42.4 | 24.0 | ND | 39.8 | ND | ND | ND | 59.9 | 24.5 | ND | 59.8 |
|  | *Escherichia coli (1070)* | ND | ND | 87.1 | 43.2 | ND | ND | ND | ND | ND | ND | ND | ND | ND | ND | ND | ND | ND | ND |
|  | *Group A Streptococcus (77)* | ND | 0.0 | 72.0 | 48.6 | ND | ND | ND | ND | ND | ND | 18.2 | ND | ND | ND | 6.1 | 18.9 | 0.0 | 75.7 |
|  | *Haemophilus influenzae (16)* | ND | ND | 68.8 | 56.3 | ND | ND | ND | ND | ND | ND | 31.3 | ND | ND | ND | ND | ND | ND | 56.3 |
|  | *Klebsiella pneumoniae (256)* | ND | ND | 81.8 | 63.3 | ND | ND | ND | ND | ND | ND | ND | ND | ND | ND | ND | ND | ND | ND |
|  | *Neisseria gonorrhoeae(18)* | ND | 0.0 | 16.7 | ND | ND | ND | ND | 0.0 | 0.0 | ND | 0.0 | ND | ND | 100.0 | 0.0 | 33.3 | ND | 100.0 |
|  | *Non-Typhoid Salmonella* spp*. (2)* | ND | ND | ND | 0.0 | ND | ND | ND | ND | ND | ND | 50.0 | ND | ND | ND | ND | 50.0 | ND | 50.0 |
|  | *Proteus* spp.(295) | ND | 80.6 | 74.8 | 48.9 | 44.0 | ND | ND | 13.1 | 12.0 | ND | 24.4 | ND | ND | ND | 68.8 | 12.0 | 66.7 | 61.4 |
|  | *Pseudomonas aeruginosa (211)* | ND | 76.9 | 95.1 | 88.9 | 0.0 | ND | ND | 13.3 | 75.0 | ND | 33.3 | ND | ND | ND | 66.4 | 14.2 | ND | 75.7 |
|  | *Staphylococcus aureus (701)* | 13.6 | 72.5 | 79.8 | 32.6 | 58.3 | ND | ND | 36.3 | 12.6 | ND | 44.4 | ND | ND | ND | 59.7 | 22.1 | 62.6 | 42.8 |
|  | *Streptococcus pneumoniae (296)* | ND | ND | 34.9 | 86.4 | ND | ND | ND | ND | ND | ND | 8.8 | ND | ND | ND | 24.2 | 8.1 | ND | 32.8 |
|  | *Salmonella typhi (39)* | ND | 69.2 | 52.2 | 10.0 | ND | ND | ND | ND | 0.0 | ND | 25.6 | ND | ND | 35.3 | 60.0 | 11.5 | ND | 38.5 |
|  | *Shigella* spp. (11) | ND | ND | 63.6 | 100.0 | ND | ND | ND | ND | ND | ND | 54.5 | ND | ND | 0.0 | 9.1 | 0.0 | ND | 0.0 |
| Kenya | *CoNS (5)* | ND | ND | 80.0 | 40.0 | 60.0 | ND | ND | ND | ND | ND | ND | 60.0 | ND | ND | 40.0 | 20.0 | ND | ND |
|  | *Escherichia coli (3062)* | ND | ND | 86.3 | 33.8 | ND | ND | ND | ND | ND | ND | ND | ND | ND | ND | ND | ND | ND | ND |
|  | *Klebsiella pneumoniae (456)* | ND | ND | ND | 50.5 | ND | ND | ND | ND | ND | ND | ND | ND | ND | ND | ND | ND | ND | ND |
|  | *Neisseria gonorrhoeae (6)* | ND | ND | ND | ND | 16.7 | ND | 0.0 | ND | ND | ND | 0.0 | ND | ND | ND | ND | 33.3 | ND | ND |
|  | *Proteus* spp. (109) | 2.9 | 65.0 | ND | 17.0 | ND | ND | ND | 1.0 | ND | 6.3 | ND | ND | ND | ND | 20.7 | 7.2 | ND | 57.1 |
|  | *Pseudomonas aeruginosa (14)* | 50.0 | ND | 50.0 | 12.5 | ND | ND | ND | ND | ND | 50.0 | 25.0 | ND | ND | ND | 20.0 | 42.9 | ND | 0.0 |
|  | *Staphylococcus aureus (816)* | 0.0 | ND | 78.3 | 9.1 | 30.0 | 0.0 | ND | ND | 10.4 | ND | 100.0 | 10.0 | ND | ND | 2.8 | 34.7 | 11.7 | 43.8 |
|  | *Streptococcus pneumoniae (55)* | ND | ND | ND | ND | ND | ND | ND | 7.3 | ND | ND | ND | ND | ND | ND | 1.8 | ND | ND | 98.2 |
|  | *Salmonella typhi (8)* | ND | ND | 75.0 | ND | ND | ND | ND | ND | ND | ND | ND | ND | ND | ND | ND | 0.0 | ND | 75.0 |
|  | *Shigella* spp*.(45)* | ND | ND | 46.7 | ND | ND | ND | ND | ND | ND | ND | ND | ND | ND | ND | ND | 4.4 | ND | 88.9 |
| Madagascar | *Escherichia coli (195)* | ND | ND | ND | 10.8 | ND | ND | ND | ND | ND | ND | ND | ND | ND | ND | ND | ND | ND | ND |
|  | *Salmonella typhi (84)* | ND | 35.7 | ND | 2.4 | ND | ND | ND | 1.2 | 0.0 | 1.2 | ND | ND | ND | 2.4 | ND | 0.0 | ND | 2.4 |
|  | *Shigella* spp. (164) | ND | 0.6 | ND | 7.3 | ND | ND | ND | 0.0 | 0.0 | 0.0 | ND | ND | ND | 8.5 | ND | 0.0 | ND | 79.9 |
| Malawi | *Group A Streptococcus (4)* | ND | ND | ND | ND | ND | ND | ND | ND | ND | ND | 0.0 | ND | ND | ND | ND | ND | ND | ND |
|  | *Group B Streptococcus (55)* | ND | ND | ND | ND | ND | ND | ND | ND | ND | ND | 0.0 | ND | ND | ND | ND | ND | ND | ND |
|  | *Haemophilus influenzae (18)* | ND | ND | ND | ND | ND | ND | ND | ND | ND | ND | 0.0 | ND | ND | ND | ND | ND | ND | ND |
|  | *Non-Typhoid Salmonella*spp*. (42)* | ND | ND | ND | ND | ND | ND | ND | ND | ND | ND | 0.0 | ND | ND | ND | ND | ND | ND | ND |
|  | *Pseudomonas aeruginosa (3)* | ND | ND | ND | ND | ND | ND | ND | ND | ND | ND | 0.0 | ND | ND | ND | ND | ND | ND | ND |
|  | *Staphylococcus aureus (17)* | ND | ND | ND | ND | ND | ND | ND | ND | ND | ND | 0.0 | ND | ND | ND | ND | ND | ND | ND |
|  | *Streptococcus pneumoniae (143)* | ND | ND | ND | ND | ND | ND | ND | ND | ND | ND | 0.0 | ND | ND | ND | ND | ND | ND | 95.8 |
| Mauritius | *Acinetobacter* spp*. (9)* | 33.3 | ND | ND | ND | ND | ND | ND | ND | ND | ND | 100.0 | ND | ND | ND | ND | ND | ND | ND |
|  | *Escherichia coli (17)* | ND | ND | 76.5 | 35.3 | ND | ND | ND | ND | ND | ND | ND | ND | ND | ND | ND | ND | ND | ND |
|  | *Haemophilus influenzae (1)* | ND | ND | 100.0 | 0.0 | ND | ND | 0.0 | ND | ND | ND | 0.0 | ND | ND | ND | ND | 0.0 | ND | ND |
|  | *Klebsiella pneumoniae(7)* | ND | ND | 100.0 | 71.4 | ND | ND | ND | ND | ND | ND | ND | ND | ND | ND | ND | ND | ND | ND |
|  | *Pseudomonas aeruginosa (8)* | 50.0 | ND | ND | ND | ND | ND | ND | ND | ND | ND | ND | ND | ND | ND | ND | ND | ND | ND |
| Mozambique | *Acinetobacter* spp*. (1)* | ND | ND | 100.0 | ND | ND | ND | ND | ND | ND | ND | 100.0 | ND | ND | ND | 100.0 | 100.0 | ND | 100.0 |
|  | *Citrobacter* spp*. (1)* | ND | ND | 100.0 | ND | ND | ND | ND | ND | ND | ND | 0.0 | ND | ND | ND | 100.0 | 0.0 | ND | 100.0 |
|  | *Escherichia coli (28)* | ND | ND | 93.0 | 64.3 | ND | ND | ND | ND | ND | ND | ND | ND | ND | ND | ND | ND | ND | ND |
|  | *Group A Streptococcus (1)* | ND | ND | 0.0 | ND | ND | ND | ND | ND | ND | ND | 0.0 | ND | ND | ND | 0.0 | 100.0 | ND | 0.0 |
|  | *Klebsiella pneumoniae (21)* | ND | ND | 100.0 | 88.2 | ND | ND | ND | ND | ND | ND | ND | ND | ND | ND | ND | ND | ND | ND |
|  | *Non-Typhoid Salmonella* spp*. (645)* | ND | ND | ND | 48.5 | ND | ND | ND | 0.0 | ND | 0.0 | 0.6 | ND | ND | ND | 47.8 | 0.2 | ND | 60.5 |
|  | *Proteus* spp. (1) | ND | ND | 0.0 | ND | ND | ND | ND | ND | ND | ND | 0.0 | ND | ND | ND | 0.0 | 0.0 | ND | 0.0 |
|  | *Pseudomonas aeruginosa (1)* | ND | ND | 100.0 | ND | ND | ND | ND | ND | ND | ND | 100.0 | ND | ND | ND | 0.0 | 0.0 | ND | 100.0 |
|  | *Staphylococcus aureus (37)* | ND | ND | 100.0 | ND | ND | ND | ND | ND | ND | ND | 47.0 | ND | ND | ND | 24.0 | 35.0 | ND | 35.0 |
|  | *Streptococcus pneumoniae (3)* | ND | ND | 33.3 | ND | ND | ND | ND | ND | ND | ND | 0.0 | ND | ND | ND | 0.0 | 100.0 | ND | 33.3 |
| Rwanda | *Acinetobacter* spp*. (22)* | 0.0 | ND | 100.0 | 100.0 | ND | ND | ND | 41.7 | ND | 44.4 | 40.0 | 50.0 | ND | ND | 61.5 | 15.9 | ND | 58.9 |
|  | *Citrobacter* spp*.(10)* | 16.7 | 83.3 | 100.0 | ND | ND | ND | ND | 18.2 | ND | 50.0 | ND | ND | ND | 80.0 | 37.5 | 10.0 | ND | 71.4 |
|  | *CoNS (99)* | 8.9 | ND | 36.7 | 25.0 | ND | ND | ND | 24.1 | ND | 38.9 | ND | 60.0 | ND | ND | 42.5 | 21.7 | ND | 58.8 |
|  | *Escherichia coli (647)* | ND | ND | 118.4 | 60.1 | ND | ND | ND | ND | ND | ND | ND | ND | ND | ND | ND | ND | ND | ND |
|  | *Klebsiella pneumoniae (201)* | ND | ND | 98.5 | 75.1 | ND | ND | ND | ND | ND | ND | ND | ND | ND | ND | ND | ND | ND | ND |
|  | *Neisseria gonorrhoeae (27)* | 60.0 | 66.7 | 50.0 | 100.0 | ND | ND | ND | 3.4 | ND | 0.0 | ND | ND | ND | ND | 20.0 | 28.1 | ND | 92.6 |
|  | *Proteus* spp. (79) | 56.8 | 83.3 | 84.6 | 66.5 | ND | ND | ND | 30.4 | ND | 30.0 | 17.0 | 57.0 | ND | ND | 85.1 | 27.5 | ND | 81.6 |
|  | *Pseudomonas aeruginosa (9)* | 14.3 | ND | 100.0 | 0.0 | ND | ND | ND | 20.0 | ND | 33.0 | ND | ND | ND | ND | 83.3 | 38.3 | ND | 83.3 |
|  | *Staphylococcus aureus (90)* | 8.0 | 30.8 | 30.8 | 20.0 | ND | ND | ND | 10.8 | ND | ND | ND | ND | ND | ND | 31.8 | 20.1 | ND | 69.0 |
|  | *Streptococcus pneumoniae (83)* | 80.0 | 35.0 | 13.0 | 0.0 | ND | ND | ND | 14.7 | ND | 20.0 | ND | ND | ND | ND | 25.6 | 40.2 | ND | 82.4 |
| Tanzania | *Acinetobacter* spp*. (15)* | ND | ND | 100.0 | 100.0 | ND | ND | ND | 100.0 | ND | 86.0 | 100.0 | ND | ND | ND | 100.0 | 47.0 | ND | 77.0 |
|  | *Citrobacter* spp*. (2)* | 0.0 | ND | 100.0 | 100.0 | ND | ND | ND | 100.0 | ND | 100.0 | 100.0 | ND | ND | ND | 0.0 | 0.0 | ND | 100.0 |
|  | *Escherichia coli (56)* | ND | ND | 100.0 | 86.9 | ND | ND | ND | ND | ND | ND | ND | ND | ND | ND | ND | ND | ND | ND |
|  | *Group B Streptococcus (28)* | ND | ND | ND | ND | ND | ND | ND | ND | ND | ND | ND | ND | ND | ND | ND | ND | 25.0 | ND |
|  | *Klebsiella pneumoniae (66)* | ND | ND | 100.0 | 94.6 | ND | ND | ND | ND | ND | ND | ND | ND | ND | ND | ND | ND | ND | ND |
|  | *Neisseria gonorrhoeae (27)* | ND | ND | ND | ND | 3.7 | ND | ND | ND | ND | ND | 0.0 | ND | ND | ND | ND | 77.8 | ND | ND |
|  | *Proteus* spp. (62) | 100.0 | ND | 85.0 | 75.7 | ND | ND | ND | 94.1 | ND | 31.8 | 32.3 | ND | ND | ND | 64.9 | 27.4 | ND | 61.3 |
|  | *Pseudomonas aeruginosa (80)* | 50.0 | ND | 100.0 | 92.9 | ND | ND | ND | ND | ND | 44.7 | 88.0 | ND | ND | ND | 100.0 | 17.4 | ND | 96.4 |
|  | *Staphylococcus aureus (138)* | 0.0 | ND | 92.4 | 17.4 | ND | ND | ND | ND | ND | ND | 11.0 | ND | ND | ND | 24.2 | 8.4 | 6.0 | 66.1 |
|  | *Salmonella typhi (8)* | 0.0 | ND | 100.0 | 0.0 | ND | ND | ND | 0.0 | ND | 0.0 | 0.0 | ND | ND | ND | 0.0 | 0.0 | ND | 0.0 |
| Uganda | *Acinetobacter* spp*. (52)* | 32.7 | ND | ND | ND | ND | ND | ND | ND | ND | 90.4 | ND | ND | ND | ND | ND | 77.0 | ND | 98.0 |
|  | *CoNS (106)* | ND | ND | 13.3 | 0.0 | ND | ND | ND | ND | ND | ND | ND | ND | ND | ND | 29.7 | 24.2 | ND | ND |
|  | *Escherichia coli (104)* | ND | ND | 221.6 | 76.3 | ND | ND | ND | ND | ND | ND | ND | ND | ND | ND | ND | ND | ND | ND |
|  | *Haemophilus influenzae(10)* | ND | ND | 100.0 | 0.0 | ND | ND | ND | ND | ND | ND | 42.9 | ND | ND | ND | 28.6 | ND | ND | 100.0 |
|  | *Klebsiella pneumoniae (45)* | ND | ND | 100.0 | 90.7 | ND | ND | ND | ND | ND | ND | ND | ND | ND | ND | ND | ND | ND | ND |
|  | *Neisseria gonorrhoeae(148)* | ND | ND | ND | ND | 0.7 | ND | ND | ND | ND | ND | 0.0 | ND | ND | ND | ND | 83.1 | ND | ND |
|  | *Proteus* spp. | 0.0 | 0.0 | 50.0 | 25.0 | 0.0 | ND | ND | 0.0 | ND | 50.0 | 0.0 | 0.0 | 100.0 | ND | 50.0 | 0.0 | ND | 75.0 |
|  | *Pseudomonas aeruginosa (15)* | 8.3 | ND | ND | ND | ND | ND | ND | ND | ND | 25.0 | ND | ND | ND | ND | ND | 16.7 | ND | 100.0 |
|  | *Staphylococcus aureus (98)* | ND | ND | 100.0 | ND | ND | ND | ND | 0.0 | ND | ND | ND | ND | ND | ND | 18.3 | 25.0 | 47.4 | 100.0 |
| Zimbabwe | *Non-Typhoid Salmonella* spp*. (127)* | ND | ND | ND | ND | ND | ND | ND | 17.3 | ND | ND | 17.3 | ND | ND | ND | 19.7 | 0.0 | ND | 34.6 |
| **Grand Total** |  | **20.6** | **59.4** | **71.3** | **47.0** | **18.3** | **66.7** | **11.9** | **31.4** | **22.1** | **37.6** | **28.1** | **47.6** | **50.0** | **36.4** | **43.2** | **25.3** | **21.8** | **66.9** |

**Continued…**

| **Country** | **Bacteria** | **do** | **e** | **cn** | **imi** | **lev** | **mer** | **met** | **na** | **nit** | **nor** | **ofl** | **ox** | **p** | **pep** | **ptz** | **tet** | **tic** | **tob** | **va** |
| --- | --- | --- | --- | --- | --- | --- | --- | --- | --- | --- | --- | --- | --- | --- | --- | --- | --- | --- | --- | --- |
| Djibouti | *Neisseria gonorrhoeae (24)* | ND | ND | ND | ND | 16.7 | ND | ND | ND | ND | ND | ND | ND | 100.0 | ND | ND | 87.5 | ND | ND | ND |
| Ethiopia | *Acinetobacter* spp*. (12)* | 41.7 | ND | 25.0 | ND | ND | ND | ND | ND | 0.0 | 25.0 | ND | ND | ND | ND | ND | ND | ND | ND | ND |
|  | *Citrobacter* spp*. (80)* | 75.8 | 83.3 | 37.5 | ND | ND | ND | ND | 42.9 | 41.2 | 25.8 | ND | 100.0 | ND | ND | ND | 50.0 | ND | ND | 100.0 |
|  | *CoNS (353)* | 54.8 | 40.9 | 22.1 | ND | ND | ND | ND | ND | 0.0 | 25.0 | ND | 57.9 | 67.4 | ND | ND | 60.9 | ND | ND | 26.9 |
|  | *Escherichia coli (1070)* | ND | ND | ND | ND | ND | ND | ND | ND | ND | ND | ND | 0.0 | 61.5 | ND | ND | ND | ND | ND | ND |
|  | *Group A Streptococcus (77)* | 50.0 | 10.4 | 24.3 | ND | ND | ND | ND | ND | ND | 24.3 | ND | 0.0 | 21.5 | ND | ND | 68.0 | ND | ND | 0.0 |
|  | *Haemophilus influenzae (16)* | 50.0 | ND | 18.8 | ND | ND | ND | ND | ND | 75.0 | 25.0 | ND | ND | 81.3 | ND | ND | 11.1 | ND | ND | ND |
|  | *Klebsiella pneumoniae (256)* | ND | ND | ND | ND | ND | ND | ND | ND | ND | ND | ND | ND | 96.3 | ND | ND | ND | ND | ND | ND |
|  | *Neisseria gonorrhoeae (18)* | ND | 16.7 | 28.6 | ND | ND | ND | ND | ND | 0.0 | ND | ND | ND | 81.8 | ND | ND | 52.9 | ND | ND | ND |
|  | *Non-Typhoid Salmonella* spp*. (2)* | 50.0 | ND | 50.0 | ND | ND | ND | ND | ND | ND | 50.0 | ND | ND | ND | ND | ND | ND | ND | ND | ND |
|  | *Proteus* spp. (295) | 64.2 | 63.6 | 26.8 | ND | ND | ND | ND | 30.8 | 56.3 | 4.4 | ND | 100.0 | 66.7 | ND | ND | 75.1 | ND | ND | 100.0 |
|  | *Pseudomonas aeruginosa (211)* | 86.9 | 77.3 | 21.7 | ND | ND | ND | ND | 92.6 | 65.9 | 17.0 | ND | 33.3 | 96.3 | ND | ND | 73.2 | ND | ND | 90.0 |
|  | *Staphylococcus aureus (701)* | 55.5 | 41.0 | 21.3 | ND | ND | ND | ND | 75.0 | 0.0 | 30.9 | ND | 68.2 | 93.8 | ND | ND | 44.8 | ND | ND | 11.8 |
|  | *Streptococcus pneumoniae (296)* | 93.3 | 26.2 | 24.5 | ND | ND | ND | ND | ND | ND | 34.0 | ND | ND | 32.5 | ND | ND | 34.5 | ND | ND | 0.0 |
|  | *Salmonella typhi (39)* | 56.3 | 100.0 | 25.6 | ND | ND | ND | ND | 15.0 | 48.3 | 56.3 | ND | ND | ND | ND | ND | 47.8 | ND | ND | ND |
|  | *Shigella* spp*. (11)* | ND | 90.9 | 27.3 | ND | ND | ND | ND | 0.0 | ND | ND | ND | ND | ND | ND | ND | 54.5 | ND | ND | ND |
| Kenya | *CoNS (5)* | 60.0 | ND | ND | ND | ND | ND | ND | ND | ND | ND | ND | 0.0 | ND | ND | ND | ND | ND | ND | 0.0 |
|  | *Escherichia coli (3062)* | ND | ND | ND | ND | ND | 0.0 | ND | ND | ND | ND | ND | ND | ND | ND | ND | ND | ND | ND | ND |
|  | *Klebsiella pneumoniae (456)* | ND | ND | ND | ND | ND | ND | ND | ND | ND | ND | ND | ND | ND | ND | ND | ND | ND | ND | ND |
|  | *Neisseria gonorrhoeae (6)* | ND | ND | ND | ND | 0.0 | ND | ND | ND | ND | ND | ND | ND | 0.0 | ND | ND | 100.0 | ND | ND | ND |
|  | *Proteus* spp *(109)* | ND | ND | 21.2 | ND | ND | 1.0 | ND | ND | ND | ND | ND | ND | ND | ND | ND | ND | ND | ND | ND |
|  | *Pseudomonas aeruginosa (14)* | ND | ND | 40.0 | 50.0 | ND | ND | ND | ND | ND | ND | ND | ND | ND | ND | 75.0 | ND | ND | ND | ND |
|  | *Staphylococcus aureus (816)* | 40.0 | 16.2 | 2.1 | ND | 1.8 | 0.0 | ND | ND | ND | ND | ND | 12.6 | 90.4 | ND | ND | 15.6 | ND | ND | 0.0 |
|  | *Streptococcus pneumoniae (55)* | ND | 3.6 | ND | ND | ND | ND | ND | ND | ND | ND | ND | ND | 9.1 | ND | ND | 0.0 | ND | ND | ND |
|  | *Salmonella typhi (8)* | ND | ND | ND | ND | ND | ND | ND | ND | ND | ND | ND | ND | ND | ND | ND | 62.5 | ND | ND | ND |
|  | *Shigella* spp*. (45)* | ND | ND | ND | ND | ND | ND | ND | ND | ND | ND | ND | ND | ND | ND | ND | 71.1 | ND | ND | ND |
| Madagascar | *Escherichia coli (195)* | ND | ND | ND | ND | ND | ND | ND | ND | ND | ND | ND | ND | ND | ND | ND | ND | ND | ND | ND |
|  | *Salmonella typhi (84)* | ND | ND | 0.0 | ND | ND | ND | ND | 1.2 | ND | ND | ND | ND | ND | ND | ND | ND | 35.7 | ND | ND |
|  | *Shigella* spp*. (164)* | ND | ND | 0.0 | ND | ND | ND | ND | 0.6 | ND | ND | ND | ND | ND | ND | ND | ND | 0.6 | ND | ND |
| Malawi | *Group A Streptococcus* | ND | 50.0 | 0.0 | ND | ND | ND | ND | ND | ND | ND | ND | ND | 0.0 | ND | ND | ND | ND | ND | ND |
|  | *Group B Streptococcus (65)* | ND | 12.5 | 100.0 | ND | ND | ND | ND | ND | ND | ND | ND | ND | ND | ND | ND | ND | ND | ND | ND |
|  | *Haemophilus influenzae 18)* | ND | ND | 5.6 | ND | ND | ND | ND | ND | ND | ND | ND | ND | ND | ND | ND | ND | ND | ND | ND |
|  | *Non-Typhoid Salmonella* spp*. (42)* | ND | ND | 11.9 | ND | ND | ND | ND | ND | ND | ND | ND | ND | ND | ND | ND | ND | ND | ND | ND |
|  | *Pseudomonas aeruginosa (3)* | ND | ND | 66.7 | ND | ND | ND | ND | ND | ND | ND | ND | ND | ND | ND | ND | ND | ND | ND | ND |
|  | *Staphylococcus aureus (17)* | ND | 0.0 | 0.0 | ND | ND | ND | ND | ND | ND | ND | ND | ND | 66.7 | ND | ND | ND | ND | ND | ND |
|  | *Streptococcus pneumoniae (143)* | ND | 0.0 | 100.0 | ND | ND | ND | ND | ND | ND | ND | ND | ND | 6.5 | ND | ND | ND | ND | ND | ND |
| Mauritius | *Acinetobacter* spp*. (9)* | ND | ND | ND | ND | 88.9 | 44.4 | ND | ND | ND | ND | ND | ND | ND | ND | 88.9 | ND | ND | ND | ND |
|  | *Escherichia coli (17)* | ND | ND | ND | ND | ND | 0.0 | ND | ND | ND | ND | ND | ND | ND | ND | 29.5 | ND | ND | ND | ND |
|  | *Haemophilus influenzae (1)* | ND | ND | ND | ND | 0.0 | 0.0 | ND | ND | ND | ND | ND | ND | ND | ND | 0.0 | ND | ND | ND | ND |
|  | *Klebsiella pneumoniae (7)* | ND | ND | ND | ND | ND | ND | ND | ND | ND | ND | ND | ND | ND | ND | ND | ND | ND | ND | ND |
|  | *Pseudomonas aeruginosa (8)* | ND | ND | ND | ND | 37.5 | 25.0 | ND | ND | ND | ND | ND | ND | ND | ND | 12.5 | ND | ND | ND | ND |
| Mozambique | *Acinetobacter* spp*. (1)* | ND | ND | 0.0 | ND | ND | ND | ND | ND | ND | ND | ND | ND | ND | ND | ND | 100.0 | ND | ND | ND |
|  | *Citrobacter* spp*. (1)* | ND | ND | 0.0 | ND | ND | ND | ND | ND | ND | ND | ND | ND | ND | ND | ND | 100.0 | ND | ND | ND |
|  | *Escherichia coli (28)* | ND | ND | ND | ND | ND | ND | ND | ND | ND | ND | ND | ND | ND | ND | 85.7 | ND | ND | ND | ND |
|  | *Group A Streptococcus (1)* | ND | ND | ND | ND | ND | ND | ND | ND | ND | ND | ND | 0.0 | 0.0 | ND | ND | 0.0 | ND | ND | 0.0 |
|  | *Klebsiella pneumoniae (21)* | ND | ND | ND | ND | ND | ND | ND | ND | ND | ND | ND | ND | ND | ND | ND | ND | ND | ND | ND |
|  | *Non-Typhoid Salmonella* spp*. (645)* | ND | ND | 18.4 | 0.0 | ND | ND | ND | 20.6 | ND | ND | 67.8 | ND | ND | ND | ND | 26.0 | ND | ND | ND |
|  | *Proteus* spp*. (1)* | ND | ND | 0.0 | 0.0 | ND | ND | ND | ND | ND | ND | ND | ND | ND | ND | ND | 0.0 | ND | ND | ND |
|  | *Pseudomonas aeruginosa (1)* | ND | ND | 0.0 | 0.0 | ND | ND | ND | ND | ND | ND | ND | ND | ND | ND | ND | 100.0 | ND | ND | ND |
|  | *Staphylococcus aureus (17)* | ND | ND | ND | ND | ND | ND | ND | ND | ND | ND | ND | 47.0 | 100.0 | ND | ND | 29.0 | ND | ND | 0.0 |
|  | *Streptococcus pneumoniae (3)* | ND | ND | ND | ND | ND | ND | ND | ND | ND | ND | ND | 0.0 | 33.3 | ND | ND | 33.3 | ND | ND | 0.0 |
| Rwanda | *Acinetobacter* spp*. (22)* | ND | ND | 31.6 | 0.0 | ND | ND | ND | 30.8 | 75.0 | 0.0 | ND | ND | ND | 33.3 | ND | 52.7 | ND | ND | ND |
|  | *Citrobacter* spp*. (10)* | ND | 100.0 | 33.3 | ND | ND | ND | ND | 41.7 | 20.0 | ND | ND | 100.0 | 100.0 | ND | ND | 40.0 | ND | ND | ND |
|  | *CoNS (99)* | ND | 36.9 | 25.0 | ND | ND | ND | ND | 85.1 | 14.7 | 67.0 | ND | 53.9 | 43.1 | ND | ND | 53.3 | ND | ND | 42.3 |
|  | *Escherichia coli (647)* | ND | ND | ND | ND | ND | ND | ND | ND | ND | ND | ND | 88.9 | 62.5 | ND | ND | ND | ND | ND | ND |
|  | *Klebsiella pneumoniae (201)* | ND | ND | ND | ND | ND | ND | ND | ND | ND | ND | ND | ND | 100.0 | ND | ND | ND | ND | ND | ND |
|  | *Neisseria gonorrhoeae (37)suda* | ND | 36.1 | 55.2 | 75.0 | ND | ND | ND | 88.5 | 28.6 | ND | ND | 87.9 | 51.4 | ND | ND | 63.6 | ND | ND | 70.0 |
|  | *Proteus* spp*.* | ND | ND | 58.0 | ND | 100.0 | ND | ND | 43.5 | 68.4 | 0.0 | 67.0 | 100.0 | ND | ND | ND | 84.3 | ND | ND | ND |
|  | *Pseudomonas aeruginosa (9)* | ND | ND | 55.6 | 100.0 | ND | ND | ND | 71.4 | 80.0 | 0.0 | ND | ND | ND | ND | ND | 100.0 | ND | ND | ND |
|  | *Staphylococcus aureus (90)* | ND | 35.5 | 20.0 | 8.3 | 0.0 | ND | ND | 84.3 | 25.5 | 33.0 | 27.0 | 73.4 | 82.6 | ND | ND | 47.2 | ND | ND | 24.6 |
|  | *Streptococcus pneumoniae (83)* | ND | 31.6 | 76.9 | ND | ND | ND | ND | 92.2 | 36.1 | ND | ND | 72.8 | 25.3 | ND | ND | 37.5 | ND | ND | 24.4 |
| Tanzania | *Acinetobacter* spp*. (15)* | ND | ND | 86.0 | ND | ND | 40.0 | ND | ND | ND | ND | ND | ND | ND | ND | ND | 73.0 | ND | ND | ND |
|  | *Citrobacter* spp*. (2)* | ND | ND | 100.0 | ND | ND | 0.0 | ND | ND | ND | 0.0 | ND | ND | ND | ND | ND | 100.0 | ND | ND | ND |
|  | *Escherichia coli (56)* | ND | ND | ND | ND | ND | 10.9 | ND | ND | ND | ND | ND | ND | ND | ND | ND | ND | ND | ND | ND |
|  | *Group B Streptococcus (28)* | ND | 12.5 | ND | ND | ND | ND | ND | ND | ND | ND | ND | ND | ND | ND | ND | 53.6 | ND | ND | ND |
|  | *Klebsiella pneumoniae (66)* | ND | ND | ND | ND | ND | ND | ND | ND | ND | ND | ND | ND | ND | ND | ND | ND | ND | ND | ND |
|  | *Neisseria gonorrhoeae (27)* | ND | ND | ND | ND | ND | ND | ND | ND | ND | ND | ND | ND | ND | ND | ND | ND | ND | ND | ND |
|  | *Proteus* spp*. (62)* | ND | ND | 34.0 | ND | ND | 1.6 | ND | ND | ND | 0.0 | ND | ND | ND | ND | ND | 94.4 | ND | ND | ND |
|  | *Pseudomonas aeruginosa (80)* | ND | ND | 34.9 | ND | ND | 25.0 | ND | ND | ND | 50.0 | ND | ND | ND | ND | ND | 100.0 | ND | ND | ND |
|  | *Staphylococcus aureus (138)* | ND | 17.0 | 28.5 | ND | ND | 0.0 | ND | ND | ND | 0.0 | ND | ND | 83.0 | ND | ND | 26.3 | ND | ND | ND |
|  | *Salmonella typhi (8)* | ND | ND | 0.0 | ND | ND | 0.0 | ND | ND | ND | 0.0 | ND | ND | ND | ND | ND | 0.0 | ND | ND | ND |
| Uganda | *Acinetobacter* spp*. (52)* | ND | ND | 88.5 | 3.9 | ND | ND | ND | ND | ND | ND | ND | ND | ND | 100.0 | 53.8 | 65.4 | ND | ND | ND |
|  | *CoNS (106)* | ND | 38.5 | 18.9 | ND | ND | ND | ND | 80.0 | ND | ND | ND | 31.9 | 20.0 | ND | ND | 56.6 | ND | ND | 0.0 |
|  | *Escherichia coli (104)* | ND | ND | ND | ND | ND | ND | ND | ND | ND | ND | ND | ND | ND | ND | 30.6 | ND | ND | ND | ND |
|  | *Haemophilus influenzae (10)* | ND | ND | 100.0 | ND | ND | ND | ND | ND | ND | ND | ND | ND | ND | ND | ND | 25.0 | ND | ND | ND |
|  | *Klebsiella pneumoniae (45)* | ND | ND | ND | ND | ND | ND | ND | ND | ND | ND | ND | ND | ND | ND | ND | ND | ND | ND | ND |
|  | *Neisseria gonorrhoeae (148)* | ND | ND | ND | ND | ND | ND | ND | ND | ND | ND | ND | ND | 68.2 | ND | ND | 97.3 | ND | ND | ND |
|  | *Proteus* spp. (2) | ND | ND | 0.0 | 0.0 | 0.0 | ND | ND | 100.0 | 100.0 | ND | ND | ND | ND | ND | ND | 100.0 | ND | ND | ND |
|  | *Pseudomonas aeruginosa (15)* | ND | ND | 16.7 | 0.0 | ND | ND | ND | ND | ND | ND | ND | ND | ND | ND | 16.7 | 100.0 | ND | ND | ND |
|  | *Staphylococcus aureus (98)* | ND | 36.8 | 21.5 | ND | ND | ND | ND | ND | ND | ND | ND | 21.3 | 78.9 | ND | ND | 68.4 | ND | ND | 0.0 |
| Zimbabwe | *Non-Typhoid Salmonella* spp*. (127)* | ND | ND | 37.0 | ND | ND | ND | ND | 16.5 | ND | ND | 44.9 | ND | ND | ND | ND | 21.3 | ND | ND | ND |
| **Grand Total** |  | **57.0** | **38.0** | **30.5** | **17.5** | **23.3** | **7.9** | **52.4** | **45.3** | **39.7** | **26.5** | **35.6** | **56.9** | **58.8** | **56.7** | **29.3** | **56.6** | **50.3** | **48.6** | **18.8** |

**A: Proportion resistance reported bacteria in East African countries**

| **Country** | **Bacteria** | **Amk** | **amx** | **amp** | **aug** | **azi** | **Cfz** | **cfp** | **ctx** | **cxt** | **caz** | **cro** | **cfu** | **cpx** | **cpt** | **c** | **cip** | **cli** | **stx** |
| --- | --- | --- | --- | --- | --- | --- | --- | --- | --- | --- | --- | --- | --- | --- | --- | --- | --- | --- | --- |
| Cameroon | *Escherichia coli (134)* | ND | ND | 20.2 | 0.0 | ND | ND | ND | ND | ND | ND | ND | ND | ND | ND | ND | ND | ND | ND |
|  | *Klebsiella pneumoniae (378)* | ND | ND | 94.0 | 32.5 | ND | ND | ND | ND | ND | ND | ND | ND | ND | ND | ND | ND | ND | ND |
|  | *Proteus* spp*. (89)* | ND | ND | 34.0 | 0.0 | ND | ND | ND | ND | ND | 0.0 | 49.4 | ND | ND | ND | 7.9 | ND | ND | 88.7 |
|  | *Pseudomonas aeruginosa (429)* | ND | ND | 92.9 | 24.4 | ND | ND | ND | ND | ND | 14.2 | 0.0 | ND | ND | ND | 67.0 | ND | ND | 100.0 |
|  | *Staphylococcus aureus (529)* | ND | ND | 95.1 | 40.0 | ND | ND | ND | ND | ND | 50.0 | 19.9 | ND | ND | ND | 63.2 | ND | ND | 100.0 |
| Congo | *CoNS (15)* | ND | ND | 60.0 | 6.7 | ND | ND | ND | 60.0 | ND | 53.3 | ND | ND | ND | ND | ND | 93.3 | ND | 66.7 |
|  | *Non-Typhoid Salmonella spp. (1094)* | ND | ND | ND | ND | 5.2 | ND | ND | 2.1 | ND | ND | ND | ND | ND | ND | 87.1 | 4.3 | ND | 88.8 |
|  | *Staphylococcus aureus (74)* | ND | ND | 78.4 | 12.2 | ND | ND | ND | 40.5 | ND | 75.0 | ND | ND | ND | ND | ND | 67.6 | 70.3 | 73.0 |
|  | *Salmonella typhi (18)* | ND | ND | 72.2 | ND | 0.0 | ND | ND | ND | ND | ND | ND | ND | ND | ND | 33.3 | ND | ND | 72.2 |
| Gabon | *Acinetobacter* spp*. (18)* | 14.3 | ND | 100.0 | 88.2 | ND | ND | ND | 100.0 | 54.5 | 77.0 | 100.0 | 100.0 | ND | ND | ND | 25.0 | ND | 80.4 |
|  | *Citrobacter* spp*. (10)* | 11.6 | 100.0 | ND | 100.0 | ND | ND | 0.0 | 45.6 | 93.7 | 13.1 | ND | ND | ND | 90.4 | ND | 31.2 | ND | 65.4 |
|  | *Escherichia coli (190)* | ND | ND | 81.7 | 61.9 | ND | ND | ND | ND | ND | ND | ND | ND | ND | ND | ND | ND | ND | ND |
|  | *Group A Streptococcus (75)* | ND | ND | 7.9 | ND | ND | ND | ND | ND | ND | ND | 0.0 | ND | ND | ND | 9.1 | ND | 7.5 | 53.7 |
|  | *Klebsiella pneumoniae (170)* | ND | ND | 100.0 | 714.3 | ND | ND | ND | 98.5 | ND | 95.5 | ND | 97.0 | ND | ND | ND | 53.7 | ND | 100.0 |
|  | *Proteus spp. (52)* | ND | ND | 34.6 | 17.3 | ND | ND | ND | ND | ND | ND | 2.1 | ND | ND | ND | 44.4 | 1.9 | ND | 48.9 |
|  | *Pseudomonas aeruginosa (39)* | ND | ND | ND | 0.0 | ND | ND | ND | ND | ND | ND | 0.0 | ND | ND | ND | ND | 2.6 | ND | ND |
|  | *Staphylococcus aureus (467)* | ND | ND | 93.8 | 7.6 | ND | ND | ND | ND | 7.6 | ND | 8.3 | ND | ND | ND | 0.8 | 8.6 | 0.0 | 20.0 |
|  | *Streptococcus pneumoniae (34)* | ND | ND | 14.8 | ND | ND | ND | ND | ND | ND | ND | 25.0 | ND | ND | ND | 22.7 | ND | 12.2 | 77.5 |
|  | *Salmonella typhi (199)* | ND | ND | 57.8 | 3.9 | 0.6 | ND | ND | ND | ND | ND | 2.9 | ND | ND | ND | 45.7 | 0.0 | ND | 50.8 |
| Sao Tome & Principe | *Staphylococcus aureus (55)* | ND | ND | ND | ND | ND | ND | ND | ND | ND | ND | ND | ND | ND | ND | 0.0 | ND | ND | ND |
| **Grand Total** |  | **20.6** | **59.4** | **71.3** | **47.0** | **18.3** | **66.7** | **11.9** | **31.4** | **22.1** | **37.6** | **28.1** | **47.6** | **50.0** | **36.4** | **43.2** | **25.3** | **21.8** | **66.9** |

**Continued…**

| **Country** | **Bacteria** | **Do** | **e** | **cn** | **imi** | **lev** | **mer** | **met** | **na** | **nit** | **nor** | **ofl** | **ox** | **p** | **pep** | **ptz** | **tet** | **tic** | **tob** | **va** |
| --- | --- | --- | --- | --- | --- | --- | --- | --- | --- | --- | --- | --- | --- | --- | --- | --- | --- | --- | --- | --- |
| Cameroon | *Escherichia coli (134)* | ND | ND | ND | ND | ND | ND | ND | ND | ND | ND | ND | 100.0 | ND | ND | ND | ND | ND | ND | ND |
|  | *Klebsiella pneumoniae (378)* | ND | ND | ND | ND | ND | ND | ND | ND | ND | ND | ND | ND | ND | ND | ND | ND | ND | ND | ND |
|  | *Proteus* spp*. (89)* | 9.4 | 7.9 | 0.0 | ND | ND | ND | ND | ND | ND | 0.0 | 0.0 | 100.0 | ND | ND | ND | ND | ND | ND | ND |
|  | *Pseudomonas aeruginosa (429)* | 85.0 | 40.8 | 29.4 | ND | ND | ND | ND | ND | ND | 0.0 | 0.0 | 100.0 | ND | ND | ND | ND | ND | ND | ND |
|  | *Staphylococcus aureus (529)* | 79.9 | 81.6 | 12.5 | ND | ND | ND | ND | ND | ND | 49.1 | 0.0 | 100.0 | ND | ND | ND | ND | ND | ND | ND |
| Congo | *CoNS (15)* | ND | 73.3 | ND | 0.0 | ND | ND | ND | ND | ND | ND | ND | ND | ND | ND | ND | ND | ND | ND | 13.3 |
|  | *Non-Typhoid Salmonella* spp*. (1094)* | ND | ND | 4.9 | ND | ND | ND | ND | 4.3 | 0.0 | ND | 90.7 | ND | ND | ND | ND | 48.2 | ND | ND | ND |
|  | *Staphylococcus aureus (74)* | ND | 68.9 | ND | 0.0 | ND | ND | ND | ND | ND | ND | ND | ND | ND | ND | ND | ND | ND | ND | 14.9 |
|  | *Salmonella typhi (18)* | ND | ND | ND | ND | ND | ND | ND | ND | ND | ND | ND | ND | ND | ND | ND | ND | ND | ND | ND |
| Gabon | *Acinetobacter* spp*. (18)* | ND | ND | 61.1 | 0.0 | ND | ND | ND | 64.0 | 60.0 | 44.0 | ND | ND | ND | 50.0 | 12.5 | ND | 64.0 | 54.5 | ND |
|  | *Citrobacter* spp*. (10)* | ND | ND | 43.7 | ND | ND | ND | ND | 62.2 | 56.0 | 38.9 | ND | ND | ND | ND | 42.3 | ND | 81.9 | 49.0 | ND |
|  | *Escherichia coli (190)* | ND | ND | ND | ND | ND | ND | ND | ND | ND | ND | ND | ND | ND | ND | 21.0 | ND | ND | 32.0 | ND |
|  | *Group A Streptococcus (75)* | ND | 7.6 | ND | ND | ND | ND | ND | ND | ND | ND | ND | ND | ND | ND | ND | ND | ND | ND | ND |
|  | *Klebsiella pneumoniae (170)* | ND | ND | 92.5 | ND | ND | 0.0 | ND | ND | ND | ND | ND | ND | ND | ND | 77.6 | ND | ND | 89.6 | ND |
|  | *Proteus* spp*. (52)* | ND | ND | 13.5 | ND | ND | ND | ND | ND | ND | ND | ND | ND | ND | ND | ND | ND | ND | ND | ND |
|  | *Pseudomonas aeruginosa (39)* | ND | ND | 0.0 | ND | ND | ND | ND | ND | ND | ND | ND | ND | ND | ND | ND | ND | ND | ND | ND |
|  | *Staphylococcus aureus (467)* | ND | 15.0 | 2.0 | ND | ND | ND | ND | ND | ND | ND | ND | 3.6 | 95.0 | ND | ND | 41.3 | ND | ND | ND |
|  | *Streptococcus pneumoniae (34)* | ND | 12.1 | ND | ND | ND | ND | ND | ND | ND | ND | ND | ND | ND | ND | ND | ND | ND | ND | ND |
|  | *Salmonella typhi (199)* | ND | ND | 0.5 | ND | ND | ND | ND | ND | ND | ND | ND | ND | ND | ND | ND | 38.2 | ND | ND | ND |
| Sao Tome & Principe | *Staphylococcus aureus (55)* | ND | ND | 0.0 | ND | ND | ND | ND | ND | ND | ND | ND | ND | ND | ND | ND | ND | ND | ND | 0.0 |
| **Grand Total** |  | **57.0** | **38.0** | **30.5** | **17.5** | **23.3** | **7.9** | **52.4** | **45.3** | **39.7** | **26.5** | **35.6** | **56.9** | **58.8** | **56.7** | **29.3** | **56.6** | **50.3** | **48.6** | **18.8** |

**B: Proportion resistance reported bacteria in Central African countries**

| **Country** | **Bacteria** | **amk** | | **amx** | | **amp** | **aug** | **azi** | **Cfz** | | **cfp** | | **ctx** | | **cxt** | | **caz** | | **cro** | | **cfu** | | **cpx** | | **cpt** | | **c** | | **cip** | | **cli** | | **stx** |  |
| --- | --- | --- | --- | --- | --- | --- | --- | --- | --- | --- | --- | --- | --- | --- | --- | --- | --- | --- | --- | --- | --- | --- | --- | --- | --- | --- | --- | --- | --- | --- | --- | --- | --- | --- |
| Egypt | *Acinetobacter* spp*. (11)* | 63.6 | | ND | | 90.9 | 100.0 | ND | ND | | ND | | 90.9 | | ND | | 90.9 | | 81.8 | | ND | | ND | | ND | | ND | | 72.7 | | ND | | ND |  |
|  | *Citrobacter* spp*. (1)* | 0.0 | | ND | | 0.0 | 0.0 | ND | ND | | ND | | 0.0 | | ND | | 0.0 | | 0.0 | | ND | | ND | | ND | | ND | | 0.0 | | ND | | ND |  |
|  | *CoNS (7)* | ND | | 28.6 | | 42.9 | ND | ND | ND | | ND | | 85.7 | | ND | | ND | | 71.4 | | ND | | ND | | ND | | ND | | 42.9 | | ND | | ND |  |
|  | *Escherichia coli (56)* | ND | | ND | | 75.2 | 43.5 | ND | ND | | ND | | ND | | ND | | ND | | ND | | ND | | ND | | ND | | ND | | ND | | ND | | ND |  |
|  | *Haemophilus influenzae (53)* | ND | | ND | | ND | 22.7 | ND | ND | | 21.4 | | ND | | ND | | ND | | ND | | ND | | ND | | ND | | ND | | 12.5 | | ND | | ND |  |
|  | *Klebsiella pneumoniae (116)* | ND | | ND | | 72.4 | 39.0 | ND | ND | | ND | | ND | | ND | | ND | | ND | | ND | | ND | | ND | | ND | | ND | | ND | | ND |  |
|  | *Proteus* spp*.(6)* | ND | | 100.0 | | ND | ND | ND | ND | | ND | | 66.7 | | ND | | ND | | 16.7 | | ND | | ND | | ND | | ND | | ND | | ND | | ND |  |
|  | *Pseudomonas aeruginosa (14)* | 34.6 | | 100.0 | | 100.0 | 100.0 | ND | ND | | ND | | 100.0 | | ND | | 100.0 | | 100.0 | | ND | | ND | | ND | | ND | | 29.0 | | ND | | ND |  |
|  | *Staphylococcus aureus (139)* | 5.1 | | 82.3 | | 80.0 | 27.0 | ND | ND | | 42.7 | | 31.2 | | ND | | ND | | 85.7 | | 52.8 | | ND | | ND | | 34.6 | | 30.7 | | 26.8 | | ND |  |
|  | *Streptococcus pneumoniae (130)* | ND | | ND | | 40.7 | 21.1 | ND | ND | | 18.0 | | ND | | ND | | ND | | ND | | ND | | ND | | ND | | ND | | ND | | ND | | ND |  |
| Libya | *Acinetobacter* spp*. (144)* | 86.1 | | ND | | 97.2 | ND | ND | 100.0 | | ND | | 93.8 | | ND | | 100.0 | | ND | | ND | | ND | | ND | | ND | | 29.9 | | ND | | ND |  |
|  | *CoNS (122)* | ND | | ND | | 100.0 | ND | ND | ND | | ND | | ND | | ND | | ND | | ND | | ND | | ND | | ND | | ND | | 59.0 | | ND | | ND |  |
|  | *Escherichia coli (315)* | ND | | ND | | 71.7 | 30.8 | ND | ND | | ND | | ND | | ND | | ND | | ND | | ND | | ND | | ND | | ND | | ND | | ND | | ND |  |
|  | *Klebsiella pneumoniae (155)* | ND | | ND | | 93.5 | 31.9 | ND | ND | | ND | | ND | | ND | | ND | | ND | | ND | | ND | | ND | | ND | | ND | | ND | | ND |  |
|  | *Pseudomonas aeruginosa (92)* | 10.9 | | ND | | ND | ND | ND | 100.0 | | ND | | 100.0 | | ND | | 7.6 | | ND | | ND | | ND | | ND | | ND | | 5.4 | | ND | | ND |  |
| Morroco | *Klebsiella pneumoniae(239)* | 50.0 | | ND | | ND | 51.0 | ND | ND | | ND | | ND | | ND | | ND | | ND | | ND | | ND | | ND | | ND | | 84.0 | | ND | | 89.0 |  |
|  | *Neisseria gonorrhoeae (72)* | ND | | ND | | ND | ND | ND | ND | | ND | | ND | | ND | | ND | | 0.0 | | ND | | ND | | ND | | ND | | 84.7 | | ND | | ND |  |
| Sudan | *CoNS (7)* | ND | | ND | | 100.0 | ND | ND | ND | | ND | | ND | | ND | | ND | | 0.0 | | ND | | ND | | ND | | ND | | ND | | ND | | ND |  |
|  | *Escherichia coli (619)* | ND | | ND | | 45.5 | 44.4 | ND | ND | | ND | | ND | | ND | | ND | | ND | | ND | | ND | | ND | | ND | | ND | | ND | | ND |  |
|  | *Klebsiella pneumoniae (9)* | ND | | ND | | 22.2 | 11.1 | ND | ND | | ND | | ND | | ND | | ND | | ND | | ND | | ND | | ND | | ND | | ND | | ND | | ND |  |
|  | *Listeria monocytogenes (21)* | ND | | ND | | 0.0 | ND | ND | ND | | ND | | ND | | ND | | ND | | 0.0 | | ND | | ND | | ND | | ND | | ND | | ND | | ND |  |
|  | *Neisseria gonorrhoeae (8)* | ND | | ND | | ND | ND | ND | ND | | ND | | 0.0 | | ND | | 25.0 | | 0.0 | | ND | | ND | | ND | | ND | | 0.0 | | ND | | 25.0 |  |
|  | *Proteus* spp*. (3)* | ND | | ND | | 33.3 | 0.0 | ND | ND | | ND | | ND | | ND | | ND | | ND | | ND | | 0.0 | | ND | | ND | | ND | | ND | | 33.3 |  |
|  | *Staphylococcus aureus (182)* | 3.0 | | 91.7 | | 81.6 | 40.9 | ND | ND | | ND | | ND | | ND | | ND | | 42.8 | | 84.2 | | ND | | ND | | 16.5 | | 54.1 | | ND | | 84.3 |  |
|  | *Salmonella typhi (17)* | 17.6 | | ND | | 23.5 | ND | ND | ND | | ND | | ND | | ND | | 0.0 | | ND | | ND | | ND | | ND | | 0.0 | | 11.8 | | ND | | ND |  |
|  | *Shigella* spp*.(36)* | 38.9 | | ND | | 44.4 | ND | ND | ND | | ND | | ND | | ND | | 22.2 | | ND | | ND | | ND | | ND | | 11.1 | | 8.3 | | ND | | ND |  |
| Tunisia | *Klebsiella pneumoniae* | 3.4 | | ND | | ND | ND | ND | ND | | ND | | ND | | ND | | ND | | ND | | ND | | ND | | ND | | 31.4 | | 57.6 | | ND | | 94.1 |  |
| **Grand Total** |  | **20.6** | | **59.4** | | **71.3** | **47.0** | **18.3** | **66.7** | | **11.9** | | **31.4** | | **22.1** | | **37.6** | | **28.1** | | **47.6** | | **50.0** | | **36.4** | | **43.2** | | **25.3** | | **21.8** | | **66.9** |  |
| **Country** | **Bacteria** | **do** | **e** | | **cn** | **imi** | **lev** | **mer** | | **met** | | **na** | | **nit** | | **nor** | | **ofl** | | **ox** | | **p** | | **pep** | | **ptz** | | **tet** | | **tic** | | **tob** | | **va** |
| Egypt | *Acinetobacter* spp*. (11)* | ND | ND | | 90.9 | 54.5 | ND | ND | | ND | | ND | | ND | | 100.0 | | ND | | ND | | ND | | 50.0 | | ND | | ND | | ND | | 50.0 | | ND |
|  | *Citrobacter spp. (1)* | ND | ND | | 0.0 | ND | ND | ND | | ND | | ND | | ND | | 0.0 | | ND | | ND | | ND | | ND | | ND | | ND | | ND | | ND | | ND |
|  | *CoNS (7)* | ND | 85.7 | | ND | 28.6 | ND | ND | | ND | | ND | | ND | | ND | | ND | | 57.1 | | ND | | ND | | ND | | ND | | ND | | ND | | 0.0 |
|  | *Escherichia coli (56)* | ND | ND | | ND | ND | ND | ND | | ND | | ND | | ND | | ND | | ND | | ND | | ND | | ND | | ND | | ND | | ND | | 42.1 | | ND |
|  | *Haemophilus influenzae (53)* | 33.0 | ND | | ND | ND | 4.6 | ND | | ND | | ND | | ND | | ND | | ND | | ND | | ND | | ND | | ND | | ND | | ND | | ND | | ND |
|  | *Klebsiella pneumoniae(116)* | ND | ND | | ND | ND | ND | ND | | ND | | ND | | ND | | ND | | ND | | ND | | ND | | ND | | ND | | ND | | ND | | ND | | ND |
|  | *Proteus* spp*.(6)* | ND | ND | | ND | 83.3 | 66.7 | ND | | ND | | ND | | ND | | ND | | ND | | ND | | ND | | ND | | ND | | ND | | ND | | ND | | 50.0 |
|  | *Pseudomonas aeruginosa (14)* | ND | ND | | 100.0 | 57.1 | 33.3 | ND | | ND | | ND | | ND | | 100.0 | | ND | | ND | | ND | | ND | | ND | | ND | | ND | | 100.0 | | 33.3 |
|  | *Staphylococcus aureus (139)* | ND | 48.2 | | 17.3 | 35.7 | 10.1 | ND | | ND | | ND | | ND | | 29.1 | | ND | | 28.1 | | ND | | ND | | ND | | 52.0 | | ND | | ND | | 3.9 |
|  | *Streptococcus pneumoniae (130)* | 41.2 | ND | | ND | ND | 5.8 | ND | | ND | | ND | | ND | | ND | | ND | | ND | | ND | | ND | | ND | | ND | | ND | | ND | | ND |
| Libya | *Acinetobacter* spp*. (144)* | ND | ND | | 68.1 | 0.0 | ND | ND | | ND | | ND | | ND | | ND | | ND | | ND | | ND | | ND | | ND | | ND | | 100.0 | | ND | | ND |
|  | *CoNS (122)* | ND | ND | | 42.6 | ND | ND | ND | | ND | | ND | | ND | | ND | | ND | | 83.6 | | ND | | ND | | ND | | 5.7 | | ND | | ND | | 1.6 |
|  | *Escherichia coli (315)* | ND | ND | | ND | ND | ND | 0.5 | | ND | | ND | | ND | | ND | | ND | | ND | | ND | | ND | | ND | | ND | | ND | | 8.2 | | ND |
|  | *Klebsiella pneumoniae (155)* | ND | ND | | ND | ND | ND | ND | | ND | | ND | | ND | | ND | | ND | | ND | | ND | | ND | | ND | | ND | | ND | | ND | | ND |
|  | *Pseudomonas aeruginosa (92)* | ND | ND | | 48.9 | 2.2 | ND | ND | | ND | | ND | | ND | | ND | | ND | | ND | | ND | | ND | | ND | | ND | | 19.6 | | ND | | ND |
| Morroco | *Klebsiella pneumoniae (239)* | ND | ND | | 89.0 | 7.0 | ND | ND | | ND | | ND | | 24.0 | | ND | | ND | | ND | | ND | | ND | | ND | | ND | | ND | | ND | | ND |
|  | *Neisseria gonorrhoeae (72)* | ND | ND | | ND | ND | ND | ND | | ND | | ND | | ND | | ND | | ND | | ND | | 55.6 | | ND | | ND | | 91.7 | | ND | | ND | | ND |
| Sudan | *CoNS (7)* | ND | ND | | 71.4 | ND | ND | ND | | 100.0 | | ND | | ND | | ND | | ND | | ND | | 100.0 | | ND | | ND | | ND | | ND | | ND | | 71.4 |
|  | *Escherichia coli* | ND | ND | | ND | ND | ND | ND | | ND | | ND | | ND | | ND | | ND | | ND | | 100.0 | | ND | | ND | | ND | | ND | | 16.5 | | ND |
|  | *Klebsiella pneumoniae (9)* | ND | ND | | ND | ND | ND | ND | | ND | | ND | | ND | | ND | | ND | | ND | | ND | | ND | | ND | | ND | | ND | | ND | | ND |
|  | *Listeria monocytogenes (21)* | ND | ND | | 0.0 | ND | ND | ND | | 4.8 | | ND | | ND | | ND | | ND | | ND | | 0.0 | | ND | | ND | | ND | | ND | | ND | | 0.0 |
|  | *Neisseria gonorrhoeae (8)* | ND | ND | | 0.0 | ND | ND | ND | | ND | | ND | | ND | | ND | | ND | | ND | | 100.0 | | ND | | ND | | ND | | ND | | ND | | ND |
|  | *Proteus* spp*. (3)* | ND | ND | | 0.0 | ND | ND | ND | | ND | | ND | | 33.3 | | ND | | ND | | ND | | ND | | ND | | ND | | ND | | ND | | ND | | ND |
|  | *Staphylococcus aureus (208)* | ND | ND | | 58.7 | 7.7 | ND | ND | | ND | | 66.2 | | 18.2 | | ND | | 51.1 | | ND | | 89.3 | | ND | | ND | | 70.0 | | ND | | ND | | 2.8 |
|  | *Salmonella typhi (17)* | ND | ND | | 0.0 | ND | ND | ND | | ND | | 23.5 | | ND | | ND | | ND | | ND | | ND | | ND | | ND | | 0.0 | | ND | | ND | | ND |
|  | *Shigella* spp*. (36)* | ND | ND | | 11.1 | ND | ND | ND | | ND | | 33.3 | | ND | | ND | | ND | | ND | | ND | | ND | | ND | | 22.2 | | ND | | ND | | ND |
| Tunisia | *Klebsiella pneumoniae* | ND | ND | | 82.2 | ND | ND | ND | | ND | | 72.0 | | ND | | ND | | ND | | ND | | ND | | ND | | ND | | ND | | ND | | 87.3 | | ND |
| **Grand Total** |  | **57.0** | **38.0** | | **30.5** | **17.5** | **23.3** | **7.9** | | **52.4** | | **45.3** | | **39.7** | | **26.5** | | **35.6** | | **56.9** | | **58.8** | | **56.7** | | **29.3** | | **56.6** | | **50.3** | | **48.6** | | **18.8** |

**C: Proportion resistance reported bacteria in North African countries**

| **Country** | **Bacteria** | **amk** | **amx** | **amp** | **aug** | **azi** | **cfz** | **cfp** | **ctx** | **cxt** | **caz** | **cro** | **cfu** | **cpx** | **cpt** | **c** | **cip** | **cli** | **stx** |
| --- | --- | --- | --- | --- | --- | --- | --- | --- | --- | --- | --- | --- | --- | --- | --- | --- | --- | --- | --- |
| Namibia | *Acinetobacter spp. (8)* | 0.0 | ND | ND | ND | ND | ND | ND | ND | ND | 16.7 | 57.1 | ND | ND | ND | ND | ND | ND | 14.3 |
|  | *Escherichia coli (7)* | ND | ND | 95.7 | 50.0 | ND | ND | ND | ND | ND | ND | ND | ND | ND | ND | ND | ND | ND | ND |
|  | *Haemophilus influenzae (55)* | ND | 34.0 | ND | 22.2 | ND | ND | ND | ND | ND | ND | 9.6 | 25.0 | ND | ND | 10.9 | 100.0 | ND | 67.6 |
|  | *Klebsiella pneumoniae (7)* | 10.0 | 100.0 | 100.0 | 39.1 | ND | ND | ND | ND | ND | 71.4 | 100.0 | 100.0 | ND | ND | ND | 0.0 | ND | 100.0 |
|  | *Neisseria meningitides* | ND | ND | ND | ND | ND | ND | ND | ND | ND | ND | 4.8 | ND | ND | ND | 2.9 | ND | ND | 77.5 |
|  | *Pseudomonas aeruginosa (7)* | 3.4 | ND | ND | ND | ND | ND | ND | ND | ND | 0.0 | ND | ND | ND | ND | ND | 0.0 | ND | 57.1 |
|  | *Staphylococcus aureus (17)* | 0.0 | 78.6 | ND | ND | ND | ND | ND | ND | ND | 50.1 | ND | 40.0 | ND | ND | ND | 19.0 | 22.2 | 62.1 |
|  | *Streptococcus pneumoniae (175)* | ND | 25.0 | ND | ND | ND | ND | ND | ND | ND | ND | 2.2 | 2.2 | ND | ND | 5.3 | 0.0 | 13.6 | 71.4 |
| South Africa | *Proteus* spp*. (23)* | 0.0 | ND | 13.0 | 0.0 | ND | ND | ND | 0.0 | ND | ND | ND | 4.3 | ND | ND | ND | 0.0 | ND | 39.1 |
|  | *Pseudomonas aeruginosa (77)* | 6.5 | ND | ND | ND | ND | ND | ND | ND | ND | 15.6 | ND | ND | ND | ND | ND | 15.6 | ND | ND |
|  | *Staphylococcus aureus (12)* | ND | ND | 91.7 | ND | ND | ND | ND | ND | ND | 25.8 | ND | ND | ND | ND | ND | 16.7 | ND | ND |
| **Grand Total** |  | **20.6** | **59.4** | **71.3** | **47.0** | **18.3** | **66.7** | **11.9** | **31.4** | **22.1** | **37.6** | **28.1** | **47.6** | **50.0** | **36.4** | **43.2** | **25.3** | **21.8** | **66.9** |

**Continued…**

| **Country** | **Bacteria** | **do** | **e** | **cn** | **imi** | **lev** | **mer** | **met** | **na** | **nit** | **nor** | **ofl** | **ox** | **p** | **pep** | **ptz** | **tet** | **tic** | **tob** | **va** |
| --- | --- | --- | --- | --- | --- | --- | --- | --- | --- | --- | --- | --- | --- | --- | --- | --- | --- | --- | --- | --- |
| Namibia | *Acinetobacter spp. (8)* | ND | ND | 25.0 | 12.5 | 0.0 | 0.0 | ND | ND | ND | ND | ND | ND | ND | 50.0 | 13.3 | ND | ND | ND | ND |
|  | *Escherichia coli (7)* | ND | ND | ND | ND | ND | 0.0 | ND | ND | ND | ND | ND | ND | 50.0 | ND | 14.8 | ND | ND | ND | ND |
|  | *Haemophilus influenzae (55)* | ND | ND | ND | ND | ND | ND | ND | ND | ND | ND | ND | ND | 0.0 | ND | ND | 25.0 | ND | ND | ND |
|  | *Klebsiella pneumoniae (7)* | ND | ND | 100.0 | ND | 0.0 | 0.0 | ND | ND | ND | ND | ND | ND | ND | ND | 20.0 | ND | ND | ND | ND |
|  | *Neisseria meningitides* | ND | 28.6 | ND | ND | ND | ND | ND | ND | ND | ND | ND | ND | 15.2 | ND | ND | 12.5 | ND | ND | ND |
|  | *Pseudomonas aeruginosa (7)* | ND | ND | 16.7 | 0.0 | 18.2 | 0.0 | ND | ND | ND | ND | ND | ND | ND | ND | 3.4 | ND | ND | ND | ND |
|  | *Staphylococcus aureus (17)* | ND | 32.3 | 52.9 | ND | ND | ND | ND | ND | ND | ND | ND | 34.5 | 73.5 | ND | ND | 29.6 | ND | ND | 0.0 |
|  | *Streptococcus pneumoniae (175)* | ND | 12.4 | 20.0 | ND | ND | ND | ND | ND | ND | ND | ND | 45.1 | 34.3 | ND | ND | 13.9 | ND | ND | 5.5 |
| South Africa | *Proteus spp. (23)* | ND | ND | 0.0 | ND | ND | ND | ND | ND | ND | ND | ND | ND | ND | ND | ND | ND | ND | ND | ND |
|  | *Pseudomonas aeruginosa (77)* | ND | ND | 18.8 | 9.8 | ND | 6.6 | ND | ND | ND | ND | ND | ND | ND | ND | 36.1 | ND | ND | 10.4 | ND |
|  | *Staphylococcus aureus (12)* | ND | 8.3 | 66.7 | ND | ND | ND | ND | ND | ND | ND | ND | 8.3 | ND | ND | ND | ND | ND | ND | ND |
| **Grand Total** |  | **57.0** | **38.0** | **30.5** | **17.5** | **23.3** | **7.9** | **52.4** | **45.3** | **39.7** | **26.5** | **35.6** | **56.9** | **58.8** | **56.7** | **29.3** | **56.6** | **50.3** | **48.6** | **18.8** |

**D: Proportion resistance reported bacteria in South African countries**

| **Country** | **Bacteria** | **amk** | **amx** | **amp** | **aug** | **azi** | **cfz** | **cfp** | **ctx** | **cxt** | **caz** | **cro** | **cfu** | **cpx** | **cpt** | **c** | **cip** | **cli** | **stx** |
| --- | --- | --- | --- | --- | --- | --- | --- | --- | --- | --- | --- | --- | --- | --- | --- | --- | --- | --- | --- |
| Benin | *Escherichia coli (84)* | ND | ND | 97.6 | 85.7 | ND | ND | ND | ND | ND | ND | ND | ND | ND | ND | ND | ND | ND | ND |
| Burkinfaso | *Escherichia coli (8)* | ND | ND | 87.5 | ND | ND | ND | ND | ND | ND | ND | ND | ND | ND | ND | ND | ND | ND | ND |
|  | *Klebsiella pneumoniae (1)* | ND | ND | 100.0 | ND | ND | ND | ND | ND | ND | ND | ND | ND | ND | ND | ND | ND | ND | ND |
|  | *Non-Typhoid Salmonella* spp*. (21)* | ND | ND | ND | ND | 0.0 | ND | ND | ND | ND | ND | ND | ND | ND | ND | 90.5 | ND | ND | 90.5 |
|  | *Salmonella typhi (37)* | ND | ND | 18.9 | ND | 0.0 | ND | ND | ND | ND | ND | ND | ND | ND | ND | 40.5 | ND | ND | 40.5 |
|  | *Shigella spp (18)* | ND | ND | 55.6 | ND | 0.0 | ND | ND | ND | ND | ND | ND | ND | ND | ND | 27.8 | 0.0 | ND | 83.3 |
| Gambia | *Salmonella typhi (10)* | ND | ND | 50.0 | ND | ND | ND | ND | ND | ND | ND | 0.0 | ND | ND | ND | 60.0 | 0.0 | ND | 60.0 |
| Ghana | *Acinetobacter* spp*. (2)* | ND | ND | 100.0 | ND | ND | ND | ND | 0.0 | ND | ND | 0.0 | 100.0 | ND | ND | 100.0 | ND | ND | 100.0 |
|  | *CoNS (27)* | ND | ND | 96.0 | ND | ND | ND | ND | ND | ND | ND | ND | 44.0 | ND | ND | ND | ND | ND | 80.0 |
|  | *Escherichia coli (6)* | ND | ND | 66.7 | ND | ND | ND | ND | ND | ND | ND | ND | ND | ND | ND | ND | ND | ND | ND |
|  | *Klebsiella pneumoniae (12)* | ND | ND | 100.0 | ND | ND | ND | ND | ND | ND | ND | ND | ND | ND | ND | ND | ND | ND | ND |
|  | *Neisseria gonorrhoeae (4)* | ND | ND | ND | ND | 50.0 | ND | ND | ND | ND | ND | 0.0 | ND | ND | ND | ND | 100.0 | ND | ND |
|  | *Non-Typhoid Salmonella* spp*. (135)* | 2.8 | ND | ND | 55.0 | ND | ND | ND | 6.1 | ND | ND | ND | 20.2 | ND | ND | 73.3 | 0.7 | ND | 62.1 |
|  | *Pseudomonas aeruginosa (58)* | ND | ND | 100.0 | ND | ND | ND | ND | 87.0 | ND | ND | 31.3 | 100.0 | ND | ND | 81.0 | ND | ND | 77.3 |
|  | *Staphylococcus aureus (310)* | ND | ND | 92.7 | ND | ND | ND | ND | ND | 1.6 | ND | ND | 55.4 | ND | ND | 64.8 | 2.2 | 0.0 | 47.4 |
|  | *Streptococcus pneumoniae (297)* | ND | ND | 0.0 | ND | ND | ND | ND | ND | ND | ND | ND | 11.0 | ND | ND | ND | ND | ND | 100.0 |
|  | *Salmonella typhi (51)* | 9.1 | ND | 68.6 | 8.3 | ND | ND | ND | 0.0 | ND | ND | 0.0 | 17.5 | ND | ND | 57.7 | ND | ND | 17.6 |
| Guinea-Bissau | *Escherichia coli (20)* | ND | ND | 50.0 | 0.0 | ND | ND | ND | ND | ND | ND | ND | ND | ND | ND | ND | ND | ND | ND |
|  | *Group A Streptococcus (1)* | ND | ND | 0.0 | ND | ND | ND | ND | 0.0 | ND | ND | ND | ND | ND | ND | 100.0 | ND | 0.0 | 0.0 |
|  | *Klebsiella pneumoniae (3)* | ND | ND | 100.0 | 66.7 | ND | ND | ND | ND | ND | ND | ND | ND | ND | ND | ND | ND | ND | ND |
|  | *Non-Typhoid Salmonella* spp*. (5)* | ND | ND | ND | 0.0 | 0.0 | ND | ND | 0.0 | ND | ND | ND | ND | ND | ND | 40.0 | 0.0 | ND | 40.0 |
|  | *Staphylococcus aureus (26)* | ND | ND | ND | ND | ND | ND | ND | 0.0 | ND | 0.0 | ND | ND | ND | ND | 0.0 | 0.0 | 0.0 | 0.0 |
|  | *Streptococcus pneumoniae (4)* | ND | ND | 0.0 | ND | ND | ND | ND | 0.0 | ND | ND | ND | ND | ND | ND | 75.0 | ND | 0.0 | 100.0 |
|  | *Salmonella typhi (3)* | ND | ND | 0.0 | 0.0 | 0.0 | ND | ND | 0.0 | ND | ND | ND | ND | ND | ND | 33.3 | 0.0 | ND | 33.3 |
| Ivorycoast | *Escherichia coli (151)* | ND | ND | ND | 37.8 | ND | ND | ND | ND | ND | ND | ND | ND | ND | ND | ND | ND | ND | ND |
| Niger | *Escherichia coli (475)* | ND | ND | ND | 7.2 | ND | ND | ND | ND | ND | ND | ND | ND | ND | ND | ND | ND | ND | ND |
|  | *Klebsiella pneumoniae (12)* | ND | ND | ND | 58.3 | ND | ND | ND | ND | ND | ND | ND | ND | ND | ND | ND | ND | ND | ND |
|  | *Salmonella typhi (404)* | 0.7 | 52.3 | ND | 16.8 | ND | ND | 12.8 | 11.4 | 0.0 | 11.4 | 0.0 | ND | ND | 6.8 | ND | 6.4 | ND | 54.7 |
|  | *Shigella spp. (154)* | 0.6 | ND | ND | 10.4 | ND | ND | ND | 0.0 | 0.0 | 0.0 | ND | ND | ND | 3.8 | ND | 0.0 | ND | 87.0 |
| Nigeria | *Acinetobacter* spp*. (9)* | ND | ND | ND | 0.0 | ND | ND | ND | ND | ND | 33.3 | 33.3 | 44.4 | ND | ND | 100.0 | 0.0 | ND | 100.0 |
|  | *Citrobacter* spp*.(33)* | ND | 73.3 | 100.0 | 0.0 | ND | ND | ND | ND | ND | 71.5 | 54.4 | ND | ND | ND | 83.3 | 42.3 | ND | 100.0 |
|  | *CONS (57)* | ND | 90.6 | 92.3 | 82.6 | ND | ND | ND | ND | 28.6 | ND | 48.0 | 71.4 | ND | ND | 69.8 | 48.7 | ND | 90.7 |
|  | *Escherichia coli (1185)* | ND | ND | 90.0 | 56.0 | ND | ND | ND | ND | ND | ND | ND | ND | ND | ND | ND | ND | ND | ND |
|  | *Group A Streptococcus (53)* | ND | 100.0 | 10.0 | 66.7 | ND | ND | ND | ND | ND | 0.0 | 14.3 | 0.0 | ND | ND | 50.0 | 10.7 | ND | 33.9 |
|  | *Haemophilus influenzae (1)* | ND | ND | 100.0 | ND | ND | ND | ND | ND | ND | 100.0 | 100.0 | ND | ND | ND | 100.0 | 16.7 | ND | 100.0 |
|  | *Klebsiella pneumoniae (687)* | ND | ND | 92.9 | 55.3 | ND | ND | ND | 11.1 | ND | 11.1 | ND | ND | ND | ND | ND | ND | ND | 88.9 |
|  | *Listeria monocytogenes (10)* | ND | ND | ND | 100.0 | ND | ND | ND | ND | ND | ND | ND | ND | ND | ND | 0.0 | ND | ND | 10.0 |
|  | *Proteus* spp*. (345)* | 16.7 | 88.6 | 91.3 | 52.8 | ND | ND | ND | 28.6 | ND | 39.9 | 38.3 | 59.9 | ND | ND | 84.0 | 38.9 | ND | 78.7 |
|  | *Pseudomonas aeruginosa (586)* | 35.8 | 92.7 | 99.5 | 75.7 | ND | ND | ND | 90.1 | ND | 45.9 | 29.9 | 93.6 | ND | ND | 47.5 | 38.8 | ND | 92.0 |
|  | *Staphylococcus aureus (1000)* | ND | 95.0 | 85.7 | 60.5 | ND | ND | ND | 100.0 | 55.1 | 30.0 | 38.0 | 45.7 | ND | ND | 82.4 | 27.2 | ND | 79.9 |
|  | *Streptococcus pneumoniae (167)* | ND | 20.0 | 68.4 | 13.8 | ND | ND | ND | ND | ND | 33.3 | 5.9 | 22.8 | ND | ND | 20.7 | 13.5 | ND | 90.4 |
|  | *Salmonella typhi (9)* | ND | 80.0 | 0.0 | 62.5 | ND | ND | ND | ND | ND | 66.7 | 25.0 | ND | ND | ND | ND | 37.5 | ND | 83.3 |
| **Grand Total** |  | **20.6** | **59.4** | **71.3** | **47.0** | **18.3** | **66.7** | **11.9** | **31.4** | **22.1** | **37.6** | **28.1** | **47.6** | **50.0** | **36.4** | **43.2** | **25.3** | **21.8** | **66.9** |

**Continued…**

| **Country** | **Bacteria** | **do** | **e** | **cn** | **imi** | **lev** | **mer** | **met** | **na** | **nit** | **nor** | **ofl** | **ox** | **p** | **pep** | **ptz** | **tet** | **tic** | **tob** | **va** |
| --- | --- | --- | --- | --- | --- | --- | --- | --- | --- | --- | --- | --- | --- | --- | --- | --- | --- | --- | --- | --- |
| Benin | *Escherichia coli (84)* | ND | ND | ND | ND | ND | ND | ND | ND | ND | ND | ND | ND | ND | ND | ND | ND | ND | ND | ND |
| Burkinfaso | *Escherichia coli (8)* | ND | ND | ND | ND | ND | 0.0 | ND | ND | ND | ND | ND | ND | ND | ND | ND | ND | ND | ND | ND |
|  | *Klebsiella pneumoniae (1)* | ND | ND | ND | ND | ND | ND | ND | ND | ND | ND | ND | ND | ND | ND | ND | ND | ND | ND | ND |
|  | *Non-Typhoid Salmonella* spp*. (21)* | ND | ND | ND | ND | ND | ND | ND | 4.8 | ND | ND | 90.5 | ND | ND | ND | ND | ND | ND | ND | ND |
|  | *Salmonella typhi (37)* | ND | ND | ND | ND | ND | ND | ND | 0.0 | ND | ND | ND | ND | ND | ND | ND | ND | ND | ND | ND |
|  | *Shigella* spp*. (18)* | ND | ND | ND | ND | ND | ND | ND | 0.0 | ND | ND | ND | ND | ND | ND | ND | ND | ND | ND | ND |
| Gambia | *Salmonella typhi (10)* | ND | ND | 20.0 | ND | ND | ND | ND | ND | ND | ND | ND | ND | ND | ND | ND | 30.0 | ND | ND | ND |
| Ghana | *Acinetobacter* spp*. (2)* | ND | ND | 0.0 | ND | ND | ND | ND | ND | ND | ND | ND | ND | ND | ND | ND | 100.0 | ND | ND | ND |
|  | *CoNS (27)* | ND | 48.0 | 28.0 | ND | ND | ND | ND | ND | ND | ND | ND | 60.0 | 96.0 | ND | ND | 76.0 | ND | ND | ND |
|  | *Escherichia coli (6)* | ND | ND | ND | ND | ND | ND | ND | ND | ND | ND | ND | ND | ND | ND | ND | ND | ND | ND | ND |
|  | *Klebsiella pneumoniae (12)* | ND | ND | ND | ND | ND | ND | ND | ND | ND | ND | ND | ND | ND | ND | ND | ND | ND | ND | ND |
|  | *Neisseria gonorrhoeae (4)* | ND | ND | ND | ND | ND | ND | ND | ND | ND | ND | ND | ND | 100.0 | ND | ND | 100.0 | ND | ND | ND |
|  | *Non-Typhoid Salmonella* spp*. (135)* | ND | ND | 9.9 | ND | ND | ND | ND | ND | ND | ND | 78.6 | ND | ND | ND | ND | 72.3 | ND | ND | ND |
|  | *Pseudomonas aeruginosa (58)* | ND | ND | 20.0 | ND | ND | ND | ND | ND | ND | ND | ND | ND | ND | ND | ND | 81.8 | ND | ND | ND |
|  | *Staphylococcus aureus (310)* | ND | 12.3 | 13.9 | ND | ND | ND | ND | ND | ND | 4.8 | ND | 19.9 | 94.9 | ND | ND | 51.0 | ND | ND | 2.8 |
|  | *Streptococcus pneumoniae (297)* | ND | 11.0 | ND | ND | ND | ND | ND | ND | ND | ND | ND | 0.0 | 0.7 | ND | ND | ND | ND | ND | ND |
|  | *Salmonella typhi (51)* | ND | ND | 4.2 | ND | ND | ND | ND | ND | ND | ND | ND | ND | ND | ND | ND | 75.6 | ND | ND | ND |
| Guinea-Bissau | *Escherichia coli (20)* | ND | ND | ND | ND | ND | ND | ND | ND | ND | ND | ND | ND | ND | ND | ND | ND | ND | ND | ND |
|  | *Group A Streptococcus (1)* | ND | 0.0 | ND | ND | ND | ND | ND | ND | ND | ND | ND | 0.0 | 0.0 | ND | ND | 0.0 | ND | ND | ND |
|  | *Klebsiella pneumoniae (3)* | ND | ND | ND | ND | ND | ND | ND | ND | ND | ND | ND | ND | ND | ND | ND | ND | ND | ND | ND |
|  | *Non-Typhoid Salmonella* spp*. (5)* | ND | ND | 0.0 | ND | ND | ND | ND | ND | ND | ND | 40.0 | ND | ND | ND | ND | ND | ND | ND | ND |
|  | *Staphylococcus aureus (26)* | ND | 0.0 | 0.0 | ND | ND | ND | ND | ND | ND | ND | ND | 0.0 | ND | ND | ND | 7.7 | ND | ND | ND |
|  | *Streptococcus pneumoniae (4)* | ND | 0.0 | ND | ND | ND | ND | ND | ND | ND | ND | ND | ND | 25.0 | ND | ND | 75.0 | ND | ND | ND |
|  | *Salmonella typhi (3)* | ND | ND | 0.0 | ND | ND | ND | ND | ND | ND | ND | ND | ND | ND | ND | ND | ND | ND | ND | ND |
| Ivorycoast | *Escherichia coli (151)* | ND | ND | ND | ND | ND | ND | ND | ND | ND | ND | ND | ND | ND | ND | ND | ND | ND | 44.2 | ND |
| Niger | *Escherichia coli (475)* | ND | ND | ND | ND | ND | ND | ND | ND | ND | ND | ND | ND | ND | ND | ND | ND | ND | ND | ND |
|  | *Klebsiella pneumoniae (12)* | ND | ND | ND | ND | ND | ND | ND | ND | ND | ND | ND | ND | ND | ND | ND | ND | ND | ND | ND |
|  | *Salmonella typhi (404)* | ND | ND | 8.2 | 0.0 | ND | ND | ND | 5.7 | ND | ND | 4.5 | ND | ND | ND | ND | ND | ND | ND | ND |
|  | *Shigella* spp*. (154)* | ND | ND | 0.6 | 0.0 | ND | ND | ND | 1.3 | ND | ND | ND | ND | ND | ND | ND | ND | ND | ND | ND |
| Nigeria | *Acinetobacter* spp*. (9)* | ND | ND | 83.3 | ND | ND | ND | ND | ND | ND | ND | 0.0 | ND | ND | ND | ND | 100.0 | ND | ND | ND |
|  | *Citrobacter* spp*. (33)* | ND | ND | 48.4 | ND | 20.0 | ND | ND | ND | 40.0 | ND | 0.0 | 100.0 | ND | ND | ND | 83.3 | ND | ND | ND |
|  | *CoNS (57)* | ND | 76.0 | 40.4 | ND | ND | ND | ND | ND | 72.2 | ND | ND | 58.6 | ND | ND | ND | 80.7 | ND | ND | 0.0 |
|  | *Escherichia coli (1185)* | ND | ND | ND | ND | ND | 20.9 | ND | ND | ND | ND | ND | 94.1 | ND | ND | 11.1 | ND | ND | ND | ND |
|  | *Group A Streptococcus (53)* | ND | 60.5 | 40.8 | ND | ND | ND | ND | ND | ND | ND | ND | 100.0 | ND | ND | ND | 50.0 | ND | ND | 0.0 |
|  | *Haemophilus influenzae(1)* | ND | 100.0 | 100.0 | ND | ND | ND | ND | ND | ND | ND | ND | 100.0 | ND | ND | ND | ND | ND | ND | ND |
|  | *Klebsiella pneumoniae 9687)* | ND | ND | ND | 11.1 | ND | ND | ND | ND | ND | ND | 44.4 | ND | ND | ND | ND | ND | ND | ND | ND |
|  | *Listeria monocytogenes (10)* | ND | 40.0 | 10.0 | ND | ND | ND | ND | ND | ND | ND | ND | 100.0 | ND | ND | ND | 60.0 | ND | ND | ND |
|  | *Proteus* spp*. (345)* | ND | 7.1 | 46.4 | 21.4 | 18.8 | 0.0 | ND | 75.0 | 32.8 | ND | 29.0 | 97.1 | ND | ND | 0.0 | 92.2 | ND | ND | ND |
|  | *Pseudomonas aeruginosa (586)* | ND | 100.0 | 35.1 | 25.9 | 15.9 | 53.0 | ND | 85.3 | 76.5 | ND | 33.2 | 100.0 | ND | ND | 0.0 | 96.2 | ND | ND | ND |
|  | *Staphylococcus aureus (1000)* | ND | 67.1 | 29.5 | ND | 18.2 | ND | ND | 71.4 | 42.5 | ND | 25.8 | 40.4 | 97.9 | ND | ND | 65.5 | ND | ND | 5.2 |
|  | *Streptococcus pneumoniae (167)* | ND | 20.0 | 10.8 | ND | 39.4 | ND | ND | ND | ND | ND | 12.9 | 50.0 | 28.0 | ND | ND | 73.7 | ND | ND | 0.0 |
|  | *Salmonella typhi (9)* | ND | ND | 22.2 | 0.0 | 40.0 | ND | ND | ND | 0.0 | ND | 40.0 | ND | ND | ND | ND | 83.3 | ND | ND | ND |
| **Grand Total** |  | **57.0** | **38.0** | **30.5** | **17.5** | **23.3** | **7.9** | **52.4** | **45.3** | **39.7** | **26.5** | **35.6** | **56.9** | **58.8** | **56.7** | **29.3** | **56.6** | **50.3** | **48.6** | **18.8** |

**E: Proportion resistance reported bacteria in West African countries**

CoNS:Coagulase Negative *Staphylococcus*spp*.*

Amk, Amikacin; Amx, Amoxicillin; Amp, Ampicillin; Aug, Amoxicillin and Clavulanic Acid; Azi, Azithromycin; Ctx, Cefotaxime; Caz, Ceftazidime; Cro, Ceftriaxone; Cfu, Cefuroxime; Cpt, Cefalotin; C, Chloramphenicol; Cip, Ciprofloxacin; Cli, Clindamycin; Sxt, Trimethoprim/Sulfamethoxazole; Do, Doxycycline; E, Erythromycin; CN, Gentamicin; Imi, Imipenem; Lev, Levofloxacin; Mer, Meropenem; NA, Nalidixic acid; Nit, Nitrofurantoin; Nor, Norfloxacin; Ofl, Ofloxacin; Ox, Oxacillin; P, Penicillin; Pip, Piperazine; PTZ, Piperacillin/tazobactam; Tet, Tetracycline; Tob, Tobramycin

ND: Not Done

Resistance >75%

Resistance 50% to 75%

Resistance 25% to 50%

Resistance below 25%
